# GDPD5-CD55-EGFR competitive binding axis regulates radioresistance and lipid accumulation in rectal cancer

**DOI:** 10.1038/s41419-026-08711-3

**Published:** 2026-04-07

**Authors:** Ruiqiu Zhu, Mingyue Li, Yi Shen, Li Zou, Limin Jin, Yuntian Shen, Yaqun Zhu, Qiliang Peng

**Affiliations:** 1 Department of Radiotherapy & Oncology, The Second Affiliated Hospital of Soochow University, Suzhou, China; 2 Department of Radiation Oncology, Suzhou Hospital, Affiliated Hospital of Medical School, Nanjing University, Suzhou, China

**Keywords:** Rectal cancer, Radiotherapy

## Abstract

Resistance to neoadjuvant chemoradiotherapy in rectal cancer diminishes survival benefits, potentially due to dysregulated lipid metabolism, though the mechanisms are unclear. Using the MSigDB database and GSE68204 cohort, we identified lipid metabolism genes linked to radiotherapy resistance. We developed resistant cell lines and xenograft models, and through multi-algorithm analysis (SVM-RFE, RF, LASSO), pinpointed key genes. Molecular mechanisms were explored via Western blotting, co-immunoprecipitation, molecular docking, and functional assays, validated in patient-derived organoids. Our study found that radiotherapy-resistant rectal cancer shows a lipid accumulation phenotype, with an inverse relationship between lipid droplet deposition and radiosensitivity in resistant cell models. The multi-algorithm screening identified GDPD5 as a key regulator. Silencing GDPD5 reduced lipid accumulation and increased radiosensitivity. Mechanistically, GDPD5 competes with CD55, disrupting its interaction with EGFR and promoting EGFR nuclear translocation, which suppresses p53 and leads to lipid buildup and radiotherapy resistance in tumors. Clinical samples showed high GDPD5 and low CD55 levels correlate with EGFR nuclear localization. Patient-derived organoids with high GDPD5 also showed increased radiotherapy resistance. Our findings indicate that GDPD5 facilitates EGFR nuclear translocation by binding to CD55, suppressing p53, and causing lipid accumulation and radiotherapy resistance in tumors. Targeting the GDPD5-CD55-EGFR interaction may enhance radiosensitivity.

## Introduction

Neoadjuvant chemoradiotherapy (nCRT) is an established therapeutic approach for rectal cancer, known for its efficacy in reducing tumor volume and recurrence rates, as well as enhancing tumor resectability and sphincter preservation rates [1–3]. Nonetheless, inadequate tumor control due to radioresistance poses a significant challenge, adversely affecting patient prognosis and survival outcomes [4]. Although advancements in radiosensitization strategies are ongoing, their clinical application remains limited [5], suggesting that the molecular mechanisms underpinning radioresistance are not yet fully understood. Notably, dysregulated lipid metabolism, characterized by abnormal lipid accumulation [6, 7] and the concomitant upregulation of fatty acid synthase (FASN) expression [8], is frequently observed in models of rectal cancer radioresistance. This lipid accumulation phenotype implies a potential link with radioresistance. However, the upstream regulatory mechanisms and the specific pathways through which it influences radioresistance have yet to be delineated.

Glycerophosphodiester Phosphodiesterase Domain Containing 5 (GDPD5), a pivotal molecule within the phospholipase D superfamily that regulates lipid metabolism [9], demonstrates biological functions across various cancers, indicating its potential role in radioresistance. In colorectal cancer, the overexpression of GDPD5 has been directly associated with tumor cell resistance to 5-fluorouracil (5-FU), and its inhibition has been shown to reverse this chemoresistance [10]. Considering that radiotherapy and 5-FU chemotherapy share similar DNA damage response mechanisms, it is plausible that the GDPD5-mediated chemoresistance pathway may similarly affect radiosensitivity. In breast cancer, GDPD5-induced dysregulation of choline phospholipid metabolism results in the accumulation of malignant phospholipid metabolites [11, 12], and such metabolic reprogramming has been independently recognized as a common characteristic of radioresistance. Moreover, research on neuroblastoma indicates that GDPD5 inhibits cell proliferation and migration through lipid metabolic pathways [13]. This modulation of malignant cell behavior may indirectly support the survival microenvironment of radioresistant cells. Taken together, current evidence suggests that GDPD5 may serve as a crucial regulator of treatment resistance via lipid metabolic reprogramming, with its regulatory network intersecting essential pathological processes associated with radioresistance.

p53, a critical mediator in the cellular response to ionizing radiation (IR), plays a pivotal role in determining cell fate post-irradiation. The suppression of p53 is associated with increased radioresistance and lipid accumulation. In head and neck squamous cell carcinoma, mutations that disrupt TP53 significantly elevate local recurrence rates following radiotherapy and contribute to radioresistance [14]. Similarly, in colorectal cancer cells, the loss of p53-binding protein 1 diminishes p53 pathway activity, thereby enhancing radioresistance [15]. Concerning lipid accumulation, p53 regulates the expression of Adipose Triglyceride Lipase (ATGL) and FASN, which leads to lipid deposition [16, 17]. In the context of radiation research, the nuclear localization of epidermal growth factor receptor (EGFR) can inhibit p53 function through direct interaction, thereby influencing radioresistance [18]. Nonetheless, the upstream mechanisms responsible for radiation-induced EGFR nuclear translocation remain poorly understood, as existing ligand-dependent pathways do not fully account for this phenomenon.

Through bioinformatic analysis and functional experiments, this study elucidates that the lipid metabolism-associated gene GDPD5 enhances radioresistance by competitively binding to CD55, thereby displacing EGFR from membrane anchoring. This displacement facilitates the nuclear translocation of EGFR, which in turn suppresses p53-dependent apoptosis and mediates cellular lipid accumulation.

## Materials and methods

### Antibodies and reagents

GDPD5 (25703-1-AP, Proteintech, AB_2880200), FASN(10624-2-AP, Proteintech, AB_2100801), ATGL(55190-1-AP, Proteintech, AB_11182818), β-actin(66009-1-Ig, Proteintech, AB_2687938), Ki67 (ab21700, Abcam, AB_446486), p53(ab241566, Abcam, AB_3675436), p53(ab32389, Abcam, AB_776981), EGFR(66455-1-Ig, Proteintech, AB_2881824), EGFR(TA506224, ZSGB-BIO, AB_2623687), CD55(82781-6-RR, Proteintech, AB_3086534), Lamin B1(12987-1-AP, Proteintech, AB_2136290), anti-Flag (66008-4-Ig, Proteintech, AB_2918475), anti-Myc (16286-1-AP, Proteintech, AB_11182162), anti-Myc (2276, Cell Signaling Technology, AB_331783), anti-HA (3724, Cell Signaling Technology, AB_1549585), anti-mouse IgG (5415, Cell Signaling Technology, AB_11112987), anti-rabbit IgG (3900, Cell Signaling Technology, AB_1550038), Nanoantibody anti Rabbit for IP (RS0121, Immunoway, AB_3732917), Nanoantibody anti Mouse for IP (RS0092, Immunoway, AB_3732925).

HEK293T, HCT116 and DLD-1 cells from Zhejiang Meisen Cell Technology Co., Ltd., China (January 2024) were authenticated and confirmed to be free of mycoplasma and contamination. HCT116/R and DLD-1/R were obtained after 25 rounds of 4 Gy irradiation.

### Ethics statement and animals

Eight-week-old male BALB/c-nu mice were purchased from Shanghai SLAC Laboratory Animal Co., Ltd. (Shanghai, China) and housed in a specific pathogen-free (SPF) animal facility at the Soochow University Animal Center. The mice were provided ad libitum access to water and standard chow and maintained on a 12-h light/dark cycle. All experimental groups included *n* = 6 mice. Randomization was strictly applied during mouse group allocation. Tumor measurements were performed by investigators blinded to group identities. All experimental procedures were conducted in compliance with the guidelines and protocols approved by the Animal Ethics Committee of Soochow University (SUDA202411A1189).

### Cell culture

HCT116 (p53-WT [19, 20]) and DLD-1 (p53-MUT S241F [20]) cells from Zhejiang Meisen Cell Technology Co., Ltd., China were authenticated and confirmed to be free of mycoplasma and contamination. The human embryonic kidney (HEK293T) cells were obtained from Beyotime Company (Shanghai, China). The cells were cultured in Dulbecco’s modified Eagle medium (DMEM) supplemented with 10% fetal bovine serum (FBS) and 1% (v/v) penicillin-streptomycin at 37 °C under 5% CO2.

### Plasmids, lentiviral vectors, shRNA, and transfection

GDPD5, CD55 and EGFR coding regions were tagged with Flag, Myc and HA, respectively, and cloned into the pCDH-CMV-MCS-EF1α-Puro vector. Lentiviral shRNA plasmids targeting USP15 were obtained from Shanghai Genechem (Shanghai, China). The sequences were as follows: shGDPD5#1 5'-GCTCCCATGATGATACCACAC-3', shGDPD5#2 5'-GCTTTACTTCTGGTGGGAAGT-3', shCD55#1 5'-GCAGTCAATGGTCAGATATTG-3', shCD55#2 CD55:5′-AUGUGAAGAAAGCUUUGUGUU-3', shP53#1 5'-GGAGCTTACCATGACCGAGTA-3', shp53#2 5′- GAAGAAAATTTCCGCAAAA-3′. Transfection of the plasmids and shRNA was performed using Lipo3000 (Invitrogen).

### Irradiation protocol

The mice were anesthetized with an intraperitoneal injection of pentobarbital sodium (approximately 40 mg/kg) and placed on a platform to receive 5 Gy of tumor using an X-RAD 320iX Biological Irradiator (Precision X-ray, North Branford, CT, USA) at a dose rate of 1.0 Gy/min. Targeting the subcutaneous tumor region was irradiated, and other parts of the body were shielded by a 5 cm-thick lead block.

Vitro model of tumor cell was established by irradiating HCT116 and DLD-1 at the dose rate of 1.0 Gy/min using an X-RAD 320iX Biological Irradiator (Precision X-ray, North Branford, CT, USA).

### RNA-Seq assay

Samples were isolated, gently washed with DEPC-treated water (10601ES76, YEASEN), frozen and then used for transcriptome RNA-Seq. Total RNA extraction, RNA integrity evaluation, library construction, and sequencing were performed according to the manufacturer’s standard protocol. RNA-seq and analysis were conducted by OE Biotech Co., Ltd. (Shanghai, China). Differentially expressed genes (DEGs) were identified using the absolute value of log2 (ratio) ≥ 1 as the threshold. The t test threshold (P values < 0.05) and fold-change threshold (> 1.5 or < 0.5) were set as the thresholds for significantly DEGs.

### Quantification of tissue triglyceride (TG) content

TG were extracted from tissue specimens through homogenization in ice-cold Reagent 1 (LE-1-145, Lai Er Bio-Tech) at a tissue mass (g)-to-reagent volume (mL) ratio of 1:5–10. Specifically, approximately 0.1 g of tissue was homogenized in 1 mL of Reagent 1 using a pre-chilled homogenizer on ice. The homogenate was centrifuged at 8000 × g for 10 min at 4 °C. The resulting supernatant was collected as the TG extract for subsequent analysis. For spectrophotometric determination, a visible light spectrophotometer was preheated for 30 min and calibrated to 505 nm wavelength.

### Colony formation assay

The tumor cells were seeded in triplicate in 6-well plates at the density of 100–2000 cells/well depending on the radiation dose. After overnight culture, followed by 0, 2, 4, 6, and 8 Gy X-ray radiation. The cells were cultured for 1–2 weeks and stained with crystal violet, and the viable colonies containing at least 50 cells were counted.

### Oil red O staining

Tissue specimens were fixed in 4% paraformaldehyde (Solarbio), cryoprotected in 30% sucrose, and embedded in OCT compound prior to cryosectioning. Cultured cells were fixed in 4% PFA for 15 min. For lipid detection, samples were stained with Oil Red O working solution for 20 min at room temperature, differentiated in 60% isopropanol, and counterstained with hematoxylin.

### BODIPY 493/503 staining

Cultured cells were fixed with 4% paraformaldehyde for 15 min at room temperature. After PBS washing, cells were incubated with BODIPY 493/503 (D3922, Thermo Fisher Scientific) for lipid droplet visualization, followed by nuclear counterstaining with DAPI (C1002, Beyotime) for 5 min. Critical precautions included avoiding antigen retrieval, detergent treatments, and cell drying throughout the procedure. Stained samples were mounted using aqueous mounting medium immediately after staining.

### Immunofluorescence (IF) staining

Cultured cells seeded in confocal dishes and cryosectioned tumor tissues (5–8 μm) underwent fixation in 4% paraformaldehyde (15 min cells/30 min tissues). Cell samples were permeabilized with 0.5% Triton X-100 (10 min), while tissue sections required antigen retrieval in sodium citrate buffer (95 °C, 15 min). All samples were blocked with 10% goat serum (1 h, RT) before incubation with primary antibodies (4 °C overnight). After PBS washes, Alexa Fluor 488/594-conjugated secondary antibodies (Thermo Fisher: A11001, A11008, A11037, A11032) were applied (1 h, RT) with DAPI counterstaining. Images were acquired using confocal microscopy (Leica) with z-stack acquisition and analyzed in LAS X software, maintaining identical exposure settings between samples.

### Mass spectrometry analysis of GDPD5 and EGFR interacting proteins

HCT116/R cells transfected with Flag-GDPD5 or Myc-EGFR were subjected to immunoprecipitation with an IgG control. Immune complexes were then incubated with Protein A/G Agarose (P2197, Beyotime). After washing four times, the immunoprecipitates were boiled in 1X loading buffer and resolved using SDS-PAGE. MS analysis was then performed by Guangzhou Saicheng Biotechnology company (Guangzhou, China).

### Immunoprecipitation (IP)

For IP analysis, cell lysis buffer (P0013, Beyotime) containing PMSF and phosphatase inhibitor cocktail was used to lyse the cells for IP analysis (P1081, Beyotime). Cell lysates were first precleared with protein A/G agarose, and then were incubated with the indicated primary antibodies at 4 °C overnight. The next day, 40 µl of protein A/G-agarose beads were added and incubated for 4 h at 4 °C. After washing three times, the mixture was resuspended. After boiling and centrifugation to pellet the agarose beads, supernatants were subjected to IB analysis.

### Co-immunoprecipitation (Co-IP) assay

Lysis buffer (Beyotime, catalog P0013), which contained a protease inhibitor cocktail (MCE, catalog HY-K0010), was used for lysis. The indicated antibodies were used in cell lysates for IP at 4 °C overnight and then incubated with protein A/G (Beyotime, catalog P2055) at 4 °C for 3 h. Then the cell lysates were washed with lysis buffer four times and then analyzed by IB.

### Rectal cancer organoid isolation and culture

Upon arrival, colorectal cancer tumor tissues were photographed and washed in the cold PBS with penicillin/streptomycin for 5 min, and then minced into tiny fragments in a sterile dish on ice. Then tissue fragments were subjected to enzymatic digestion (K601003, BioGenous) in 10 mL digestion medium containing 9.5 mL basal medium (K601003-A100, BioGenous) at 37 °C for 30–60 min. Tumor organoids were collected after centrifugation at 300 g for 5 min. The resuspended organoids were subsequently mixed with an equal volume of Matrigel® (356231, Corning) and then seeded on a prewarmed 24-well plate at a density of 500 crypts per well. Colorectal cancer organoid complete medium (K2103-CR; BioGenous) (500 μL) was then added to each well. The organoids were treated with complete medium with or without 100 μM I3A 1 h pre-IR and then exposed to 6 Gy X-rays at a dose rate of 1.1 Gy/min or sham-irradiated. The organoids were finally viewed under an optical microscope, followed by analysis of the organoids using ImageJ software. At least 50 organoids were counted. The study received approval from the Clinical Research Ethics Committee of the Second Affiliated Hospital of Soochow University (Approval Number: JD-LK2022167-IR02).

### Analysis of the lipid metabolism gene set

Using the MSigDB database to find related gene sets in hallmark, KEGG, and Reactome, collectively termed LMRGs. The GSE68204 dataset from the GEO database, comprising 59 rectal cancer patients undergoing preoperative NCRT, was analyzed. Patients were categorized into NCRT responders (27) and non-responders (32) based on pathology results. The raw GSE68204 data were processed using the Affy package in R, and differential expression was analyzed with the “Limma” package, using a P value < 0.05. The DEGs were cross-referenced with LMRGs to identify DE-LMRGs, whose ability to distinguish between the two NCRT groups was evaluated using principal component analysis (PCA). Functional enrichment analyses, including GO and KEGG analysis, were conducted to understand the biological role of DE-LMRGs. The R software “ClusterProfiler” and “ggplot2” packages were used for analysis and visualization. Candidate LMRGs were identified using LASSO, SVM, and RF machine learning algorithms. Their accuracy in predicting NCRT for rectal cancer was evaluated with ROC curves.

### Statistical analysis

Analyses were carried out using GraphPad Prism software (Version 7.0). Data are represented as the mean ± SD. Two-tailed Student’s t-tests and one-way ANOVAs were utilized in the data analysis for the article. Cohen’s d for estimating effect size. A P-value less than 0.05 was considered to be statistically significant.

## Results

### Radiotherapy resistance in rectal cancer is linked to lipid accumulation

**Fig. 1:**
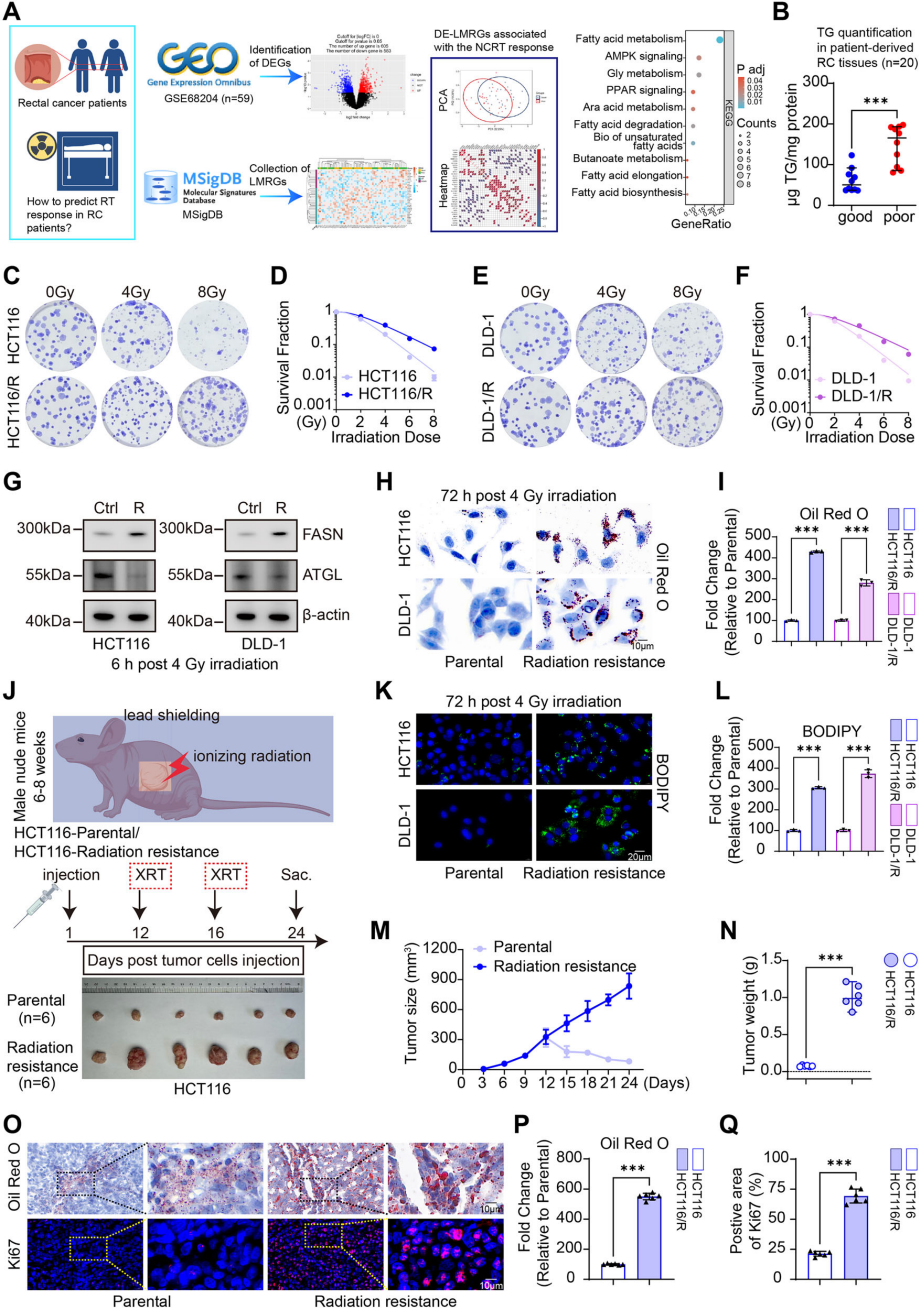
Lipid accumulation associates with rectal cancer radioresistance. **A** Schematic of bioinformatics analysis, The GSE68204 dataset was integrated with the MSigDB to conduct differential gene screening, PCA, correlation analysis, and KEGG enrichment analysis. **B** Assessment of triglyceride levels in tumor tissues from rectal cancer patients (*n* = 20), categorized by treatment response (good response, TRG 0-1; poor response, TRG 2-3). **C–F** Clonogenic assays and statistical analyses were performed on both parental and radioresistant HCT116 and DLD-1 cell lines. **G** Western blot analysis was conducted to determine the protein levels of FASN and ATGL in parental and radioresistant HCT116 and DLD-1 cell lines. **H**, **I** Oil Red O staining (scale bar = 10 μm) and quantification of neutral lipids were performed in parental and radioresistant HCT116 and DLD-1 cell lines. **J** Schematic representation of the nude mouse subcutaneous tumor model and images of gross tumors (*n* = 6 per group). **K**, **L** BODIPY staining (scale bar = 20 μm) and quantification of neutral lipids were conducted in parental and radioresistant (R) HCT116 and DLD-1 cell lines. **M** Growth curves of subcutaneous tumors derived from parental and radioresistant cell lines were plotted (*n* = 6 per group, *P* (24 days) <0.001, Cohen’s d (24 days)=7.91). **N** Statistical analysis of tumor weights was performed comparing parental and radioresistant groups (*n* = 6 per group, Cohen’s d = 8.67). **O–Q** Oil Red O staining (*n* = 6 per group, Cohen’s d = 28.53) and Ki67 IF analysis (*n* = 6 per group, Cohen’s d = 11.03), along with statistical evaluations (scale bar = 10 μm), were conducted in parental and radioresistant groups. NS indicates non-significance, **p < 0.01; ***p < 0.001.

To elucidate the mechanisms underlying radioresistance in rectal cancer, we conducted an initial screening of the MSigDB database, identifying 763 genes associated with lipid metabolism related genes (LMRGs). Transcriptomic analysis of the GSE68204 cohort revealed that, in comparison to radiotherapy responders (27 patients), non-responders (32 patiens) exhibited 583 upregulated and 605 downregulated differentially expressed genes (DEGs). An intersection analysis of these datasets with the LMRGs identified 36 differentially expressed LMRGs. PCA demonstrated a significant separation in LMRG expression profiles between the two groups. Interaction network analysis revealed strong associations among these genes, and KEGG pathway enrichment further indicated their collective involvement in the regulation of lipid metabolism (Fig. 1A). Validation using clinical samples showed significantly elevated lipid content in tumor tissues from non-responders (*n* = 10) compared to radiotherapy responders (*n* = 10) among 20 rectal cancer patient tissues (Fig. 1B).

Subsequently, we successfully developed radioresistant models using HCT116 and DLD-1 cell lines (Fig. 1C–F). Western blot (WB) analysis revealed an upregulation of FASN and a downregulation of ATGL expression in the radioresistant cell lines (Fig. 1G). Oil Red O staining and BODIPY fluorescence staining demonstrated significant lipid accumulation in the radioresistant cells (Fig. 1H, I, K, L). In vivo experiments employing a subcutaneous xenograft tumor model indicated that tumors derived from radioresistant cells exhibited reduced sensitivity to IR compared to those derived from parental cells (Fig. 1J, M, N), while also showing an increased Ki67-positive rate and enhanced histological lipid deposition (Fig. 1O–Q). Overexpression of FASN in parental cell lines induces lipid accumulation and radiation resistance (Fig. S1A–E), while inhibiting lipid synthesis in radioresistant cell line induces radiosensitization (Fig. S1F–I). Collectively, these findings confirm that the radioresistant phenotype in rectal cancer is significantly associated with tumor lipid accumulation.

### GDPD5 modulates lipid accumulation and radiosensitivity in rectal cancer

**Fig. 2:**
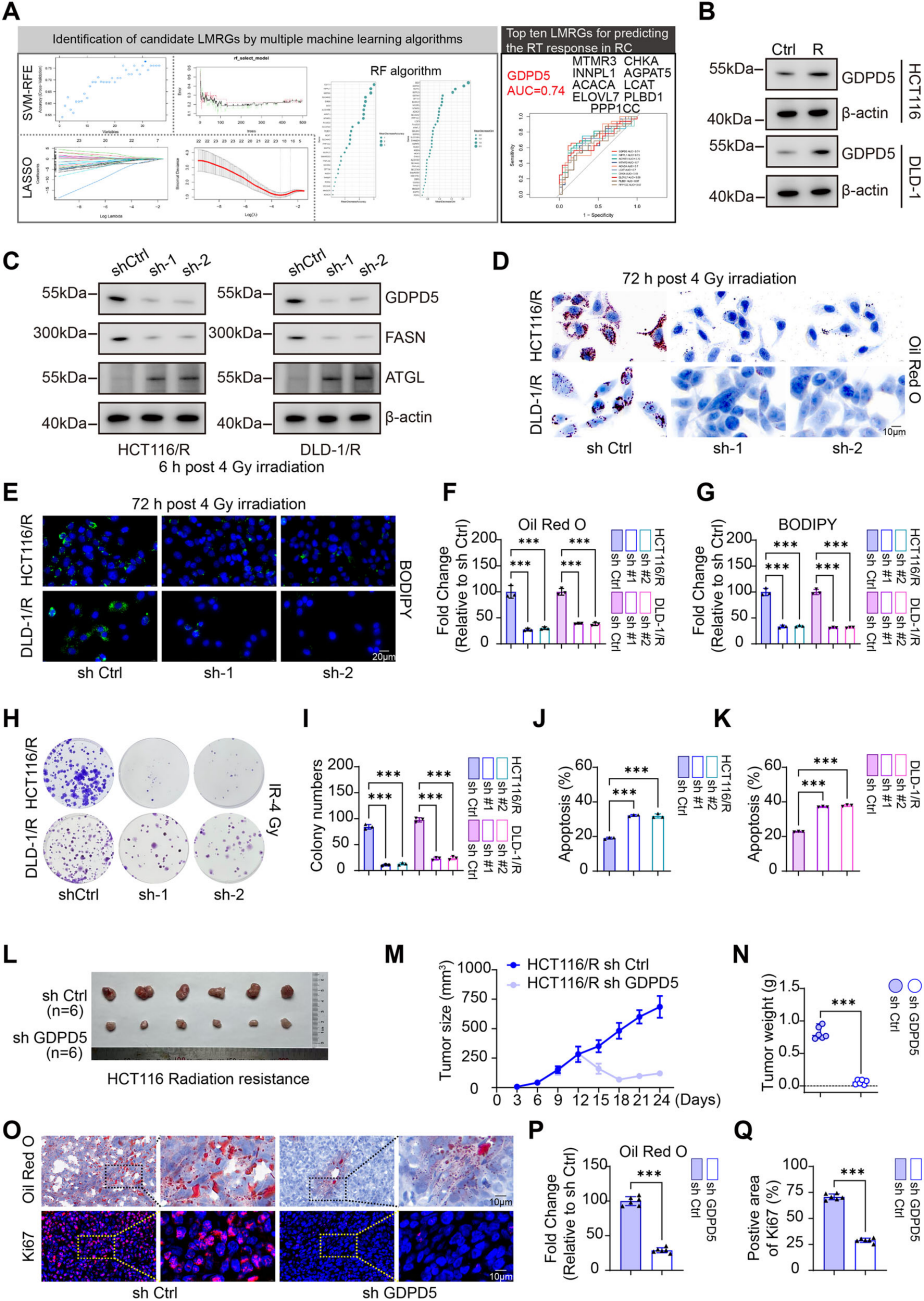
GDPD5 regulates lipid accumulation and radiosensitivity in rectal cancer cells. **A** Screening of core lipid metabolism genes was conducted using SVM-RFE, RF, and LASSO logistic regression, followed by ROC curve analysis to evaluate the AUC values of core genes. **B** WB analysis was performed to detect GDPD5 protein levels in both parental and radioresistant HCT116 and DLD-1 cell lines. **C** WB analysis was also utilized to assess the protein levels of GDPD5, FASN, and ATGL in control versus GDPD5-knockdown radioresistant cell lines. **D**, **F** Oil Red O staining (scale bar = 10 μm) and quantification of neutral lipids were conducted in control and GDPD5-knockdown radioresistant cells. **E**, **G** BODIPY staining (scale bar = 20 μm) and neutral lipid quantification were performed in control and GDPD5-knockdown radioresistant cells. **H**, **I** Clonogenic assays (IR-4 Gy) and statistical analyses were conducted in control and GDPD5-knockdown radioresistant cells. **J**, **K** Flow cytometry was employed to detect apoptosis, with statistical analysis performed on irradiated (IR-4 Gy) control and GDPD5-knockdown radioresistant cells. **L** Gross tumor images were captured for control and GDPD5-knockdown HCT116 radioresistant cells (*n* = 6 per group). **M** Tumor growth curves were generated to compare radioresistant control and GDPD5-knockdown groups (*n* = 6 per group, *P* (24 days) <0.001, Cohen’s d (24 days)=8.44). **N** Tumor weight statistics for radioresistant control vs. GDPD5-knockdown groups (*n* = 6 per group, Cohen’s d = 10.75). **O–Q** Oil Red O staining (*n* = 6 per group, Cohen’s d = 14.11) and Ki67 IF detection (*n* = 6 per group, Cohen’s d = 17.32) in radioresistant control and GDPD5-knockdown groups (Scale bar=10 μm). sh #1, shGDPD5-1; sh #2, shGDPD5-2. NS non-significance, **p < 0.01; ***p < 0.001.

To elucidate the regulatory mechanisms connecting lipid accumulation with radioresistance, this study utilized support vector machine recursive feature elimination (SVM-RFE), random forest (RF), and LASSO logistic regression algorithms to identify differentially expressed LMRGs, resulting in the identification of 10 core regulatory genes. Receiver operating characteristic (ROC) curve analysis indicated that all top 10 key LMRGs exhibited area under the curve (AUC) values exceeding 0.67, thereby confirming their predictive stability. Among these genes, GDPD5 demonstrated the most significant predictive efficacy for chemoradiotherapy response and was identified as the primary candidate gene (Fig. 2A). WB analysis showed that GDPD5 expression was significantly elevated in radioresistant cell lines compared to their parental counterparts (Fig. 2B). The knockdown of GDPD5 expression in radioresistant cells resulted in a significant downregulation of FASN and an upregulation of ATGL protein levels (Fig. 2C) with these metabolic alterations and associated radioresistance phenotypes being completely reversed by re-expression of shRNA-resistant GDPD5 constructs (Fig. S2C–G). Oil Red O staining and BODIPY fluorescence staining demonstrated that GDPD5 knockdown effectively mitigated the lipid accumulation phenotype in radioresistant cells (Fig. 2D–G). Clonogenic survival assays and flow cytometric analysis of apoptosis further revealed that GDPD5 knockdown significantly augmented IR-induced clonogenic cell death and apoptosis (Fig. 2H–K). GDPD5 was overexpressed in HCT116 and DLD-1 cell lines, resulting in increased lipid accumulation and enhanced resistance to radiation therapy (Fig. S3). In vivo experiments corroborated that GDPD5 knockdown increased the radiosensitivity of radioresistant cells to IR while simultaneously reducing lipid accumulation in tumor tissues (Fig. 2L–Q). Collectively, these findings identify GDPD5 as a critical molecular regulator of lipid accumulation and radiosensitivity in rectal cancer.

### GDPD5 modulates lipid accumulation and radiosensitivity through the p53 pathway

**Fig. 3:**
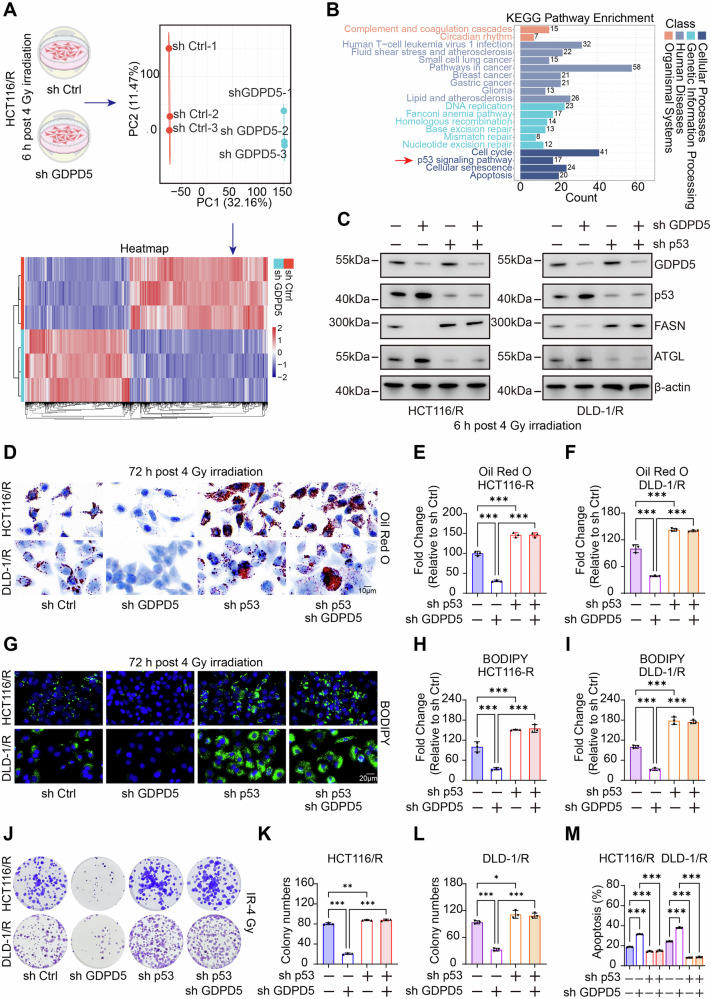
GDPD5 modulates lipid accumulation and radiosensitivity via the p53 signaling pathway. **A** PCA and Heatmap illustrating differentially expressed genes from RNA-seq analysis comparing control and GDPD5-knockdown HCT116 radioresistant cells. **B** KEGG pathway enrichment analysis of differentially expressed genes from RNA-seq in control versus GDPD5-knockdown HCT116 radioresistant cells. **C** WB analysis of GDPD5, p53, FASN, and ATGL protein levels across different groups in radioresistant cells. **D–F** Oil Red O staining (Scale bar=10 μm) and quantification of neutral lipids across groups. **G–I** BODIPY staining (Scale bar=20 μm) and quantification of neutral lipids across groups. **J–L** Clonogenic assays following irradiation (IR-4 Gy) and statistical analysis across groups. **M** Flow cytometry-based apoptosis detection and statistical analysis following irradiation (IR-4 Gy) across groups. NS not significant, **p < 0.01; ***p < 0.001.

To investigate how GDPD5 influences lipid accumulation and radiosensitivity, we created a GDPD5-knockdown model in radioresistant HCT116 cells. We conducted transcriptome sequencing on samples taken 6 h after 4 Gy irradiation from both control and knockdown groups. PCA showed clear separation between the two groups. GDPD5 deficiency led to the upregulation of 256 genes and downregulation of 250 genes compared to controls (Fig. 3A). KEGG enrichment analysis of these DEGs revealed significant enrichment in the p53 signaling pathway (Fig. 3B). Moreover, previous studies have substantiated the regulatory role of the p53 pathway in modulating ATGL and FASN expression. Knockdown of p53 mediates lipid accumulation and reduced radiosensitivity (Fig. S4). WB, Oil Red O staining, and BODIPY fluorescence staining revealed that the reduction in lipid accumulation, induced by GDPD5 knockdown in radioresistant cell lines, was completely reversed by simultaneous p53 knockdown (Fig. 3C–I). Furthermore, clonogenic survival assays and flow cytometric analysis of apoptosis corroborated that the enhanced radiosensitizing effect, resulting from GDPD5 knockdown, was also negated by p53 knockdown (Fig. 3J–M). Collectively, these functional rescue assays demonstrate that GDPD5 modulates lipid accumulation and radiosensitivity in rectal cancer via a p53-dependent pathway.

### The interaction complex of GDPD5, CD55, and EGFR

**Fig. 4:**
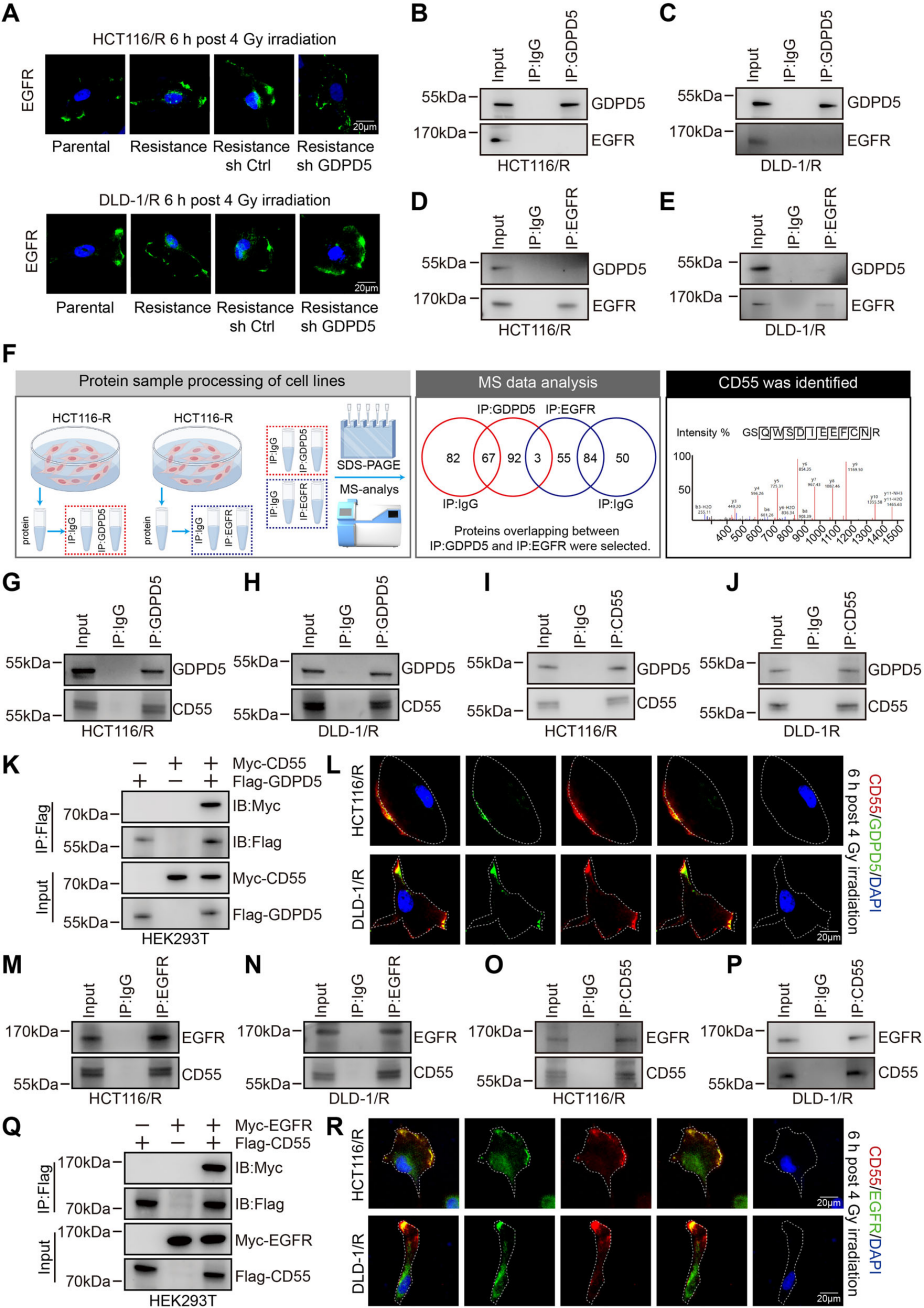
Protein interactions among GDPD5, CD55, and EGFR. **A** IF analysis was conducted to examine the distribution of EGFR across different groups (Scale bar = 20 μm). **B–E** Co-IP analysis was performed using antibodies against GDPD5 and EGFR in radioresistant cells. **F** A schematic representation of the mass spectrometry analysis is provided, following Co-IP using GDPD5 or EGFR as baits in HCT116 radioresistant cells, with subsequent identification of intersecting interacting proteins. **G–J** Co-IP analysis was conducted using antibodies against GDPD5 and CD55 in radioresistant cells. **K** Co-IP analysis of GDPD5-CD55 interactions was performed in HEK293T cells, utilizing an anti-Flag antibody for Flag-GDPD5 and an anti-Myc antibody for Myc-CD55. **L** IF analysis was conducted to assess the co-localization of GDPD5 and CD55 in radioresistant cells (Scale bar = 20 μm). **M–P** Co-IP analysis was performed using antibodies against EGFR and CD55 in radioresistant cells. **Q** Co-IP analysis of EGFR-CD55 interactions was conducted in HEK293T cells, employing an anti-Flag antibody for Flag-CD55 and an anti-Myc antibody for Myc-EGFR. **R** IF analysis was conducted to evaluate the co-localization of EGFR and CD55 in radioresistant cells (Scale bar = 20 μm).

Previous research has demonstrated that p53, a pivotal effector molecule involved in the regulation of radiosensitivity, is modulated by the nuclear translocation of EGFR. In the present study, IF assays indicated an enhanced nuclear localization of EGFR in radioresistant cell lines compared to their parental counterparts, with a marked reduction in this localization following GDPD5 knockdown (Fig. 4A, S6). This observation led us to hypothesize that GDPD5 may influence p53-dependent radiosensitivity through the regulation of EGFR. However, Co-IP assays revealed no direct interaction between GDPD5 and EGFR (Fig. 4B–E). To further investigate this indirect regulatory mechanism, we conducted Co-IP coupled with mass spectrometry for both GDPD5 and EGFR to identify common interacting proteins. Bioinformatics intersection analysis identified CD55 as a potential interactor with both proteins (Fig. 4F). Subsequent Co-IP and IF co-localization assays confirmed that CD55 specifically interacts with both GDPD5 and EGFR (Fig. 4G–R). These findings collectively demonstrate that GDPD5, CD55, and EGFR form an interaction complex.

### CD55-mediated competitive binding of GDPD5 and EGFR regulates EGFR nuclear translocation

**Fig. 5:**
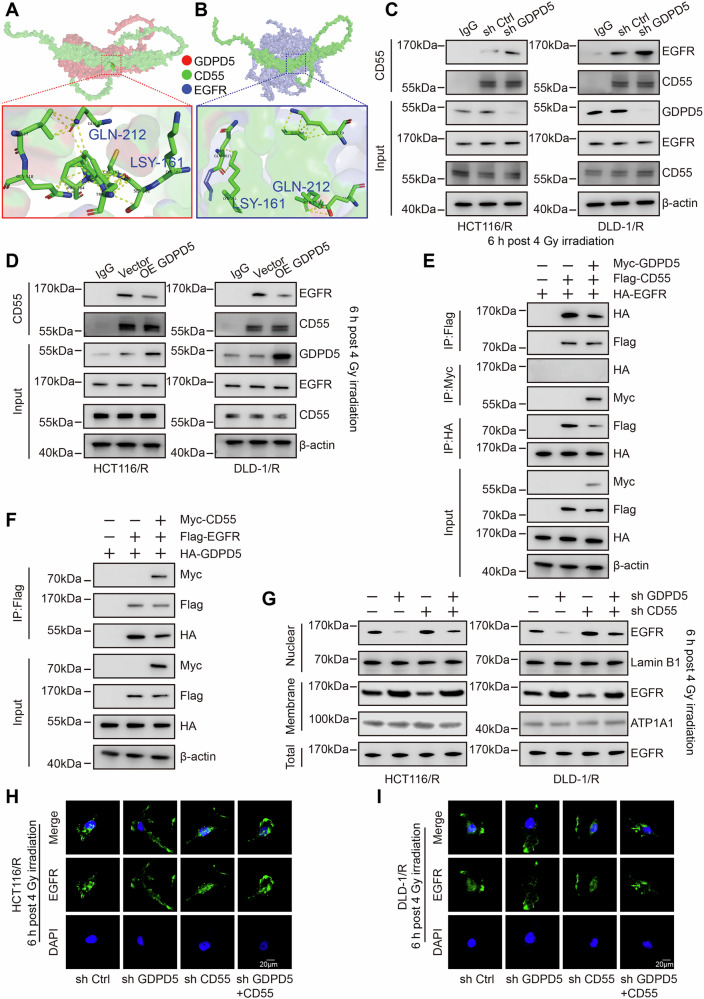
CD55 facilitates competitive binding between GDPD5 and EGFR, thereby influencing the nuclear translocation of EGFR. **A** Molecular docking model illustrating the interaction between GDPD5 and CD55. **B** Molecular docking model depicting the interaction between EGFR and CD55. **C**, **D** Co-IP using a CD55 antibody to assess the binding levels of EGFR and CD55 in cells with GDPD5 knockdown (**C**) and in cells overexpressing GDPD5 (**D**) that exhibit radioresistance. **E** Co-IP analysis of GDPD5-CD55-EGFR interactions in HEK293T cells, utilizing an anti-Myc antibody for Myc-tagged GDPD5, an anti-Flag antibody for Flag-tagged CD55, and an anti-HA antibody for HA-tagged EGFR. **F** Co-IP analysis of EGFR interactions in HEK293T cells, employing an anti-HA antibody for HA-tagged GDPD5, an anti-Myc antibody for Myc-tagged CD55, and an anti-Flag antibody for Flag-tagged EGFR. **G** Western blot analysis detecting nuclear, membrane, and total EGFR levels across different groups in radioresistant cells. **H**, **I** IF detection of EGFR distribution in radioresistant cells (Scale bar = 20 μm).

To elucidate the regulatory mechanism underlying the GDPD5-CD55-EGFR interaction complex, molecular docking simulations have demonstrated that the LYS-161 and GLN-212 sites of CD55 are capable of binding both GDPD5 and EGFR. This finding suggests the potential for competitive binding of CD55 between these two proteins (Fig. 5A, B). In radioresistant cell lines, alterations in GDPD5 expression levels, in conjunction with Co-IP assays, have confirmed that the binding affinity between CD55 and EGFR is contingent upon GDPD5 expression levels (Fig. 5C, D). This competitive binding model has been further substantiated by in vitro IP assays (Fig. 5E, F). To assess the functional implications of this interaction on EGFR subcellular localization, WB and IF analyses have shown that GDPD5 knockdown results in the suppression of EGFR nuclear translocation. Notably, simultaneous knockdown of CD55 under these conditions partially mitigates this suppression of EGFR nuclear translocation (Fig. 5G–I). The evidence suggests that CD55, functioning as a membrane-anchored protein, sequesters EGFR at the plasma membrane through direct binding, thereby inhibiting its translocation to the nucleus. In contrast, the interaction of GDPD5 with CD55 facilitates the nuclear translocation of EGFR.

### GDPD5 modulates lipid accumulation and radiosensitivity via a CD55-dependent mechanism

**Fig. 6:**
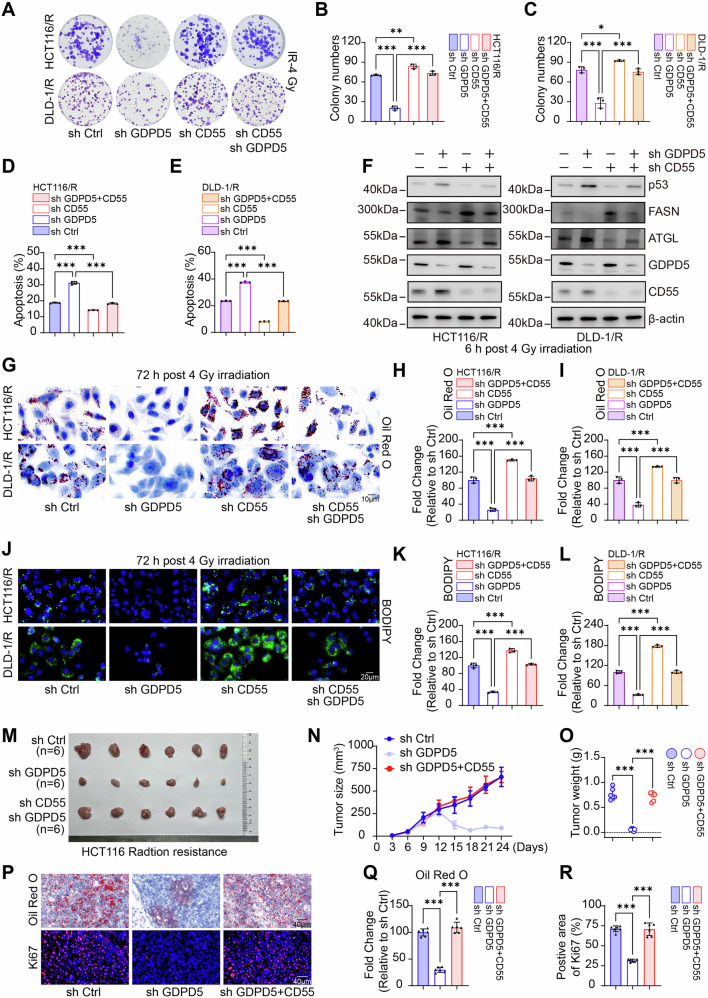
The regulation of lipid accumulation and radiosensitivity by GDPD5 is contingent upon CD55. **A–C** Clonogenic assays were conducted using IR at a dose of 4 Gy, with statistical analyses performed across different groups of radioresistant cells. **D, E** Flow cytometry was utilized for apoptosis detection following IR exposure (4 Gy), accompanied by statistical evaluations across groups. **F** WB analysis was employed to detect the expression levels of GDPD5, CD55, p53, FASN, and ATGL across the various groups. **G–I** Oil Red O staining was performed to visualize neutral lipids (Scale bar = 10 μm), and statistical analyses were conducted across groups. **J–L** BODIPY staining was similarly executed (Scale bar = 20 μm), and neutral lipid quantification was assessed across groups. **M** Gross tumor images were obtained from control, GDPD5-knockdown, and GDPD5/CD55 dual-knockdown HCT116 radioresistant cells (*n* = 6 per group). **N** Tumor growth curves were plotted for the different groups (*n* = 6 per group, *P* (sh Ctrl vs sh GDPD5, 24 days) <0.001, Cohen’s d (sh Ctrl vs sh GDPD5, 24 days) =7.33, *P* (sh GDPD5 + CD55 vs sh GDPD5, 24 days) <0.001, Cohen’s d (sh GDPD5 + CD55 vs sh GDPD5, 24 days) =12.22). **O** Tumor weight measurements were statistically analyzed across groups (*n* = 6 per group, Cohen’s d (sh Ctrl vs sh GDPD5) = 8.79, Cohen’s d (sh GDPD5 + CD55 vs sh GDPD5) = 10.45). **P–R** Oil Red O staining (*n* = 6 per group, Cohen’s d (sh Ctrl vs sh GDPD5) = 11.99, Cohen’s d (sh GDPD5 + CD55 vs sh GDPD5) = 10.20) and Ki67 IF detection were conducted (*n* = 6 per group, Cohen’s d (sh Ctrl vs sh GDPD5) = 13.63, Cohen’s d (sh GDPD5 + CD55 vs sh GDPD5) = 6.90), and statistical analyses were performed across groups (Scale bar = 40 μm). NS indicates non-significance, **p < 0.01; ***p < 0.001.

To elucidate the regulatory function of the GDPD5-CD55 interaction complex on lipid accumulation and radiosensitivity, we first confirmed CD55-specific functional phenotypes using two distinct shRNAs (Fig. S5A), with full phenotypic rescue by shRNA-resistant CD55 constructs establishing target specificity (Fig. S5G–K). We then conducted functional rescue experiments by silencing CD55 in GDPD5-knockdown radioresistant cell lines. Our findings demonstrated that the increased radiosensitivity resulting from GDPD5 knockdown was reversed upon simultaneous CD55 knockdown (Fig. 6A–E). Concurrently, the decrease in lipid accumulation associated with GDPD5 deficiency was also mitigated by CD55 knockdown (Fig. 6F–L). In vivo subcutaneous xenograft tumor models further corroborated that the radiosensitizing effects induced by GDPD5 knockdown, evidenced by suppressed tumor growth, a reduced Ki67-positive rate, and diminished lipid accumulation—were effectively counteracted by CD55 knockdown (Fig. 6M–R). Collectively, these findings suggest that GDPD5 modulates lipid metabolism and radiosensitivity in rectal cancer through a CD55-dependent pathway.

### Clinical significance of the GDPD5-CD55-EGFR competitive binding axis and integrated mechanism

**Fig. 7:**
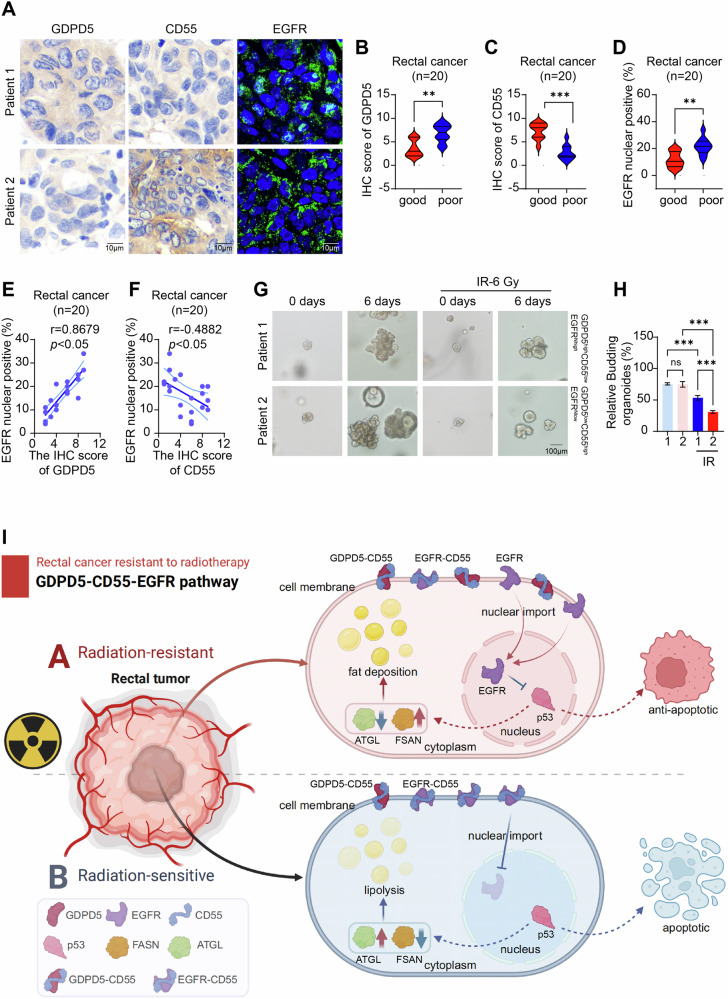
Clinical significance of the GDPD5-CD55-EGFR axis in rectal cancer. **A** Immunohistochemical analysis of GDPD5, CD55, and EGFR expression in rectal cancer tissue samples (*n* = 20), accompanied by IHC scoring (**B–D**). **E**, **F** Correlation analysis of the IHC findings. **G** Representative images depicting tumor organoid models comparing sham and IR-6 Gy treatment groups. **H** Statistical analysis of the organoid response to radiation. **I** Graphical abstract illustrating the mechanism by which GDPD5 competitively binds to CD55 and EGFR, facilitating EGFR nuclear translocation, leading to the suppression of p53 and the promotion of radioresistance and lipid accumulation. NS denotes non-significance, **p < 0.01; ***p < 0.001.

To ascertain the clinical significance of the GDPD5-CD55-EGFR competitive binding axis, immunohistochemical analyses were conducted on patient tissue samples. The findings indicated that patients who responded favorably to radiotherapy exhibited low levels of GDPD5 expression, elevated CD55 expression, and decreased nuclear localization of EGFR (Fig. 7A–D). Subsequent correlation analysis revealed a positive association between the number of EGFR nuclear-positive cells and GDPD5 expression, alongside a negative association with CD55 expression (Fig. 7E, F). Furthermore, these findings were statistically significant in both p53 wild-type and mutant patient tissue samples (Fig. S7). Utilizing these results, isogenic patient tissues were employed to develop patient-derived organoid (PDO) models with high GDPD5 expression, low CD55 expression, high EGFR nuclear localization and low GDPD5 expression, high CD55 expression, low EGFR nuclear localization (Fig. S8A). The GDPD5-high PDO group displayed enhanced radioresistance, as demonstrated by significantly increased post-irradiation organoid budding rate and area, elevated IC50, and extended half-maximal proliferation time (Fig. 7G, H, S8B–E).

In conclusion, this study introduces a mechanistic model in which GDPD5 regulates the nuclear translocation of EGFR by competitively binding to the membrane-anchored protein CD55, thereby affecting p53-mediated lipid metabolic reprogramming and radiosensitivity. The findings underscore the pivotal role of the GDPD5-CD55-EGFR competitive binding axis in promoting radioresistance in rectal cancer and offer a theoretical basis for developing targeted therapeutic interventions (Fig. 7I).

## Discussion

Radiotherapy is a fundamental therapeutic approach for different stages of rectal cancer. However, its effectiveness is often limited by the radioresistance of tumor cells. This study elucidates the molecular mechanism by which the GDPD5-CD55-EGFR competitive binding complex influences p53-associated lipid accumulation and simultaneously promotes radioresistance through the regulation of EGFR nuclear translocation. The concurrent occurrence of lipid accumulation within tumor tissue and radioresistance is frequently documented. However, the underlying causal relationship remains inadequately elucidated. Clinical observations indicate elevated expression of CPT1A, a critical enzyme in fatty acid oxidation, in radioresistant nasopharyngeal carcinoma and pancreatic cancer tissues [21], accompanied by significantly reduced serum levels of high-density lipoprotein cholesterol [22, 23]. Multi-omics analyses further reveal aberrant accumulation of membrane phospholipids and arachidonic acid precursors, along with persistently increased lipid droplet content within radioresistant tumors. Notably, FASN overexpression is associated with enhanced DNA repair capacity in radioresistant cells [24], while cholesterol accumulation is observed concurrently with Wnt pathway activation [23]. Our findings also suggest that patients with rectal cancer who exhibit no response to radiotherapy demonstrate significantly increased intratumoral lipid content compared to responders. Furthermore, a lipid accumulation phenotype is consistently observed in both radioresistant cell lines and subcutaneous xenograft models. Furthermore, it was discovered in cellular models that lipid content influences radiosensitivity. The findings of this study indicate that GDPD5-CD55-EGFR-p53 axis functions as a higher-order regulatory mechanism. The knockdown of GDPD5 concurrently mitigates lipid accumulation, reinstates p53-dependent apoptotic processes, and impedes the nuclear translocation of EGFR, thereby collectively counteracting radioresistance. Consequently, although lipid accumulation is a requisite element, GDPD5 acts as the principal orchestrator of this phenotype.

Subsequent investigations indicate that this process may be modulated by GDPD5. Prior research has demonstrated the tumor-promoting role of GDPD5 in solid tumors, with significant overexpression observed in colorectal cancer tissues. In these tissues, GDPD5 facilitates metastasis and induces resistance to 5-FU by promoting epithelial-mesenchymal transition [10]. In breast cancer, GDPD5 enhances tumor cell migration through the hydrolysis of glycerophosphocholine (GPC) [25]. Although its potential to induce differentiation has been documented in neuroblastoma [26], GDPD5 is located within the frequently deleted chromosomal region 11q13 [27]. This deletion is directly associated with poor prognosis in advanced-stage neuroblastoma, implying that the tumor-suppressive function of GDPD5 may be context-dependent. Importantly, the oncogenic mechanisms of GDPD5 closely align with its role in transmembrane signaling regulation. As a glycosylphosphatidylinositol (GPI)-anchored hydrolase, the loss of its membrane localization results in a decreased release of GPI-anchored proteins [28]. In contrast, the overexpression of GDPD5 in colorectal cancer may activate oncogenic pathways, exemplified by the GDPD5-CD55-EGFR competitive binding complex identified in this study, potentially through the stabilization of membrane-localized complexes [10]. This reliance on membrane localization is apparent in both its hydrolytic activity towards GPC and its role in the regulation of lipid metabolism [25]. Taken together, the aforementioned evidence offers a mechanistic explanation for GDPD5’s involvement in modulating radioresistance and lipid accumulation in rectal cancer.

This study elucidates that the regulation of GDPD5 is contingent upon the p53 signaling pathway. Serving as a pivotal node in metabolic regulation, the functional inhibition of p53 results in a significant lipid accumulation phenotype [29]. p53 facilitates transcriptional activation by binding to the ATGL promoter. Consequently, the absence or functional inhibition of p53 leads to impaired triglyceride hydrolysis [16]. Additionally, the USP22-FASN axis remains constitutively active in p53-deficient cells, promoting excessive lipid accumulation and tumor progression [17]. Beyond ATGL and FASN, p53 deficiency may also augment the expression of lipogenic genes through the derepression of SREBP-1c and suppress lipolytic pathways by downregulating ADRB3, further intensifying lipid accumulation [29, 30]. Importantly, p53-mediated metabolic reprogramming demonstrates considerable tissue and stress specificity [29]. In the context of radioresistance, this reprogramming, potentially through alterations in lipid metabolic phenotypes, may affect DNA damage repair efficiency and cell survival [31]. Moreover, as a fundamental regulator of radiosensitivity, the functional status of p53 directly influences tumor response to radiotherapy. Patients with bladder cancer and head and neck squamous cell carcinoma that retain functional p53 exhibit significantly improved complete response rates to radiotherapy [32]. The underlying mechanism entails the protein p53 coordinating the cellular response to radiation via a bifurcated program. Initially, it activates the p21-mediated G1/S phase arrest, thereby providing an opportunity for DNA repair. Subsequently, it triggers the PUMA/BAX-dependent apoptotic pathway to eliminate cells with irreparable damage. [33, 34]. When p53 function is compromised, tumor cells not only lose their ability to undergo radiation-induced apoptosis but also experience dysregulation of the G2/M checkpoint, resulting in a marked increase in clonogenic survival [35]. Collectively, these insights provide the theoretical basis for the findings in this study, which demonstrate that GDPD5, acting through the p53 pathway, regulates lipid accumulation and radioresistance in rectal cancer.

TP53 mutations are present in 50–70% of colorectal cancers and primarily consist of missense mutations, truncating mutations (nonsense and frameshift), and less frequently, splice site and regulatory region mutations [36, 37]. Missense mutations are the most prevalent and may retain partial functions, such as phosphorylation and pro-apoptotic activity, as well as “gain-of-function” properties [38, 39]. In contrast, truncating mutations and splice site mutations typically result in a complete loss of function [40–42]. Regulatory region mutations result in downregulation of p53 function [36]. This study demonstrates that the GDPD5-CD55-EGFR axis operates in a p53-context-dependent manner. In cells with wild-type p53, this axis fully regulates lipid metabolism and radiosensitivity through p53. In models with missense mutations, silencing GDPD5 continues to suppress lipid accumulation and enhance radiosensitivity, effects that can be reversed by co-knockdown of CD55, thereby confirming the core functionality of the axis with partially active p53. However, in models with truncating and splice site mutations, the axis is anticipated to be ineffective, as changes in lipid accumulation and radiosensitivity are contingent upon p53 functionality. Future research should undertake a systematic comparison of responses across various mutation types, namely missense, truncating, splice site, and regulatory mutations, within isogenic backgrounds. Additionally, efforts should be directed towards the development of p53 phosphorylation enhancers specifically for missense mutants, as well as the design of combination therapies that operate independently of p53 for truncating or null mutations.

In radioresistant models, the modulation of p53 activity is influenced by the process of EGFR nuclear translocation, which constitutes a critical mechanism underlying tumor radioresistance. Upon exposure to IR, EGFR is transported retrogradely via the Golgi-endoplasmic reticulum pathway and subsequently translocated through the nuclear pore complex, facilitated by the coordinated actions of the Sec61 translocon and importin β1 [43]. Clinical studies indicate that EGFR nuclear translocation in rectal cancer patients following radiotherapy is significantly associated with an elevated risk of local recurrence [44]. Once translocated to the nucleus, EGFR suppresses p53 transcriptional activity by promoting the formation of a complex with DNA-PKcs, thereby inhibiting its pro-apoptotic function [18]. This interaction is contingent upon EGFR-mediated enhancement of DNA-PKcs binding and results in the attenuation of p53 stability by preventing phosphorylation at its Ser15 residue [18]. Moreover, research has demonstrated that the Wnt/EGFR pathway activates EGFR signaling through rhomboid, which subsequently suppresses the Chk2/p53-dependent DNA damage response pathway to IR, thereby reducing apoptosis [45]. Importantly, EGFR inhibitors have been shown to restore p53 activity [18]. Our study identifies an increase in EGFR nuclear translocation in radioresistant rectal cancer models. Significantly, GDPD5 was observed to affect this EGFR nuclear translocation, a finding corroborated by analyses of patient-derived tissues. Consequently, we propose that GDPD5 may modulate the p53 pathway by regulating EGFR nuclear translocation.

We have determined that there is no direct binding relationship between GDPD5 and EGFR. CD55, which functions as a GPI-anchored membrane glycoprotein, exerts its non-canonical tumor-suppressive effects through molecular scaffolding facilitated by its short consensus repeat (SCR) domains. These domains are crucial for maintaining the homeostasis of key receptors within lipid raft microdomains. Previous research has shown that CD55 negatively regulates oncogenic pathways via its SCR domains, binding to CD97 suppresses excessive JAK/STAT3 activation, while interaction with the LIME/ROR2 complex limits LCK/JNK signaling intensity [46–48]. These mechanisms are implicated in the dysregulation of DNA repair associated with radioresistance. In this study, we identify residues LYS-161 and GLN-212 within CD55 as the specific binding sites for EGFR. The competitive occupancy of this site by GDPD5 disrupts the CD55-EGFR interaction and promotes the nuclear translocation of EGFR. This process consequently inhibits the function of p53, promotes the accumulation of lipids, and ultimately results in radioresistance. This mechanism provides novel evidence supporting the tumor-suppressive role of CD55. Research indicates that the depletion of CD55-positive cells is associated with increased radioresistance [49], potentially due to unchecked nuclear trafficking of EGFR. In contrast, the expression of CD55 induced by hypoxia-inducible factor 1-alpha within hypoxic microenvironments may delay the onset of resistance by stabilizing the localization of EGFR at the membrane [50]. This observation is consistent with clinical findings that high CD55 expression is associated with improved prognosis in patients with gastric cancer [46]. Therapeutically restoring the CD55-EGFR interaction presents significant potential. Enhancing the function of CD55 in cervical cancer models activates caspase-3-dependent apoptosis [51]. Additionally, this study confirms that inhibiting GDPD5 competition enhances CD55-mediated anchoring of EGFR to the membrane. By maintaining p53 expression, this intervention reverses lipid accumulation and radioresistance.

The causal relationship between lipid accumulation and radioresistance observed in this study necessitates further investigation, as current evidence primarily highlights their co-occurrence. Previous research suggests that lipid droplets may sequester free radicals, thereby mitigating oxidative damage [6, 52]. Additionally, enhanced fatty acid synthesis in radioresistant tumor cells facilitates membrane phospholipid biogenesis and the release of signaling molecules [53]. Elevated expression of ATP citrate lyase (ACLY) is associated with poor prognosis in patients undergoing radiotherapy, while inhibition of ACLY impairs DNA damage repair capacity [54]. Increased expression and activity of FASN have been observed in radioresistant tumors [55]. FASN upregulates the androgen receptor via the Akt/NF-κB pathway, leading to cell cycle arrest and enhanced DNA repair [56]. The key enzyme in fatty acid oxidation, carnitine palmitoyltransferase 1A (CPT1A), enhances DNA damage repair and suppresses apoptosis by modulating FOXM1 [57]. It is overexpressed in radioresistant patients and negatively correlates with survival rates [21]. The DGAT2 inhibitor PF-06424439, which targets a lipid droplet metabolic enzyme, reduces lipid droplet accumulation and sensitizes cells to radiation [58]. These findings collectively suggest that the reprogramming of lipid metabolism contributes to radioresistance by providing essential membrane components, energy, and substrates necessary for repair.

Recent research underscores the pivotal role of feedback regulation between p53 and lipid metabolism in tumor progression. The accumulation of lipid droplets facilitates the anchoring of p53 to the lipid droplet surface through interactions with cytochrome b5 reductase 3 and myosin heavy chain 9 [59, 60]. This anchoring renders p53 vulnerable to specific ubiquitination and subsequent degradation by the E3 ubiquitin ligase MDM2 [60]. The deficiency of p53 subsequently liberates ribosomal protein S3A, promoting its nuclear translocation in complex with the transcription factor C/EBPβ [61]. This complex enhances the expression of peroxisome proliferator-activated receptor gamma and the fatty acid transporter CD36, resulting in sustained lipid droplet expansion. This deleterious cycle—where lipid droplet accumulation triggers p53 degradation, and the loss of p53 facilitates de novo lipogenesis—has received further clinical validation [62].

Future research should focus on inducing lipid accumulation in radiation-sensitive rectal cancer cell lines to evaluate the resulting alterations in radiotherapeutic response. Moreover, the therapeutic potential of targeting CD55 warrants further investigation. Although this study demonstrated that CD55 knockdown intensifies radioresistance, the translational significance of CD55 as a tumor suppressor must be validated through overexpression models. Employing CRISPR activation technology or lipid nanoparticle-mediated delivery of CD55 mRNA could provide insights into whether these approaches can counteract GDPD5-mediated EGFR nuclear translocation and radioresistance [63, 64]. For clinical translation, the integration of radiomics to assess intratumoral lipid content, such as quantifying lipid droplet distribution via chemical shift imaging, could serve as an indirect biomarker for evaluating rectal cancer radiosensitivity [65, 66].

In conclusion, GDPD5 competitively interacts with the LYS-161/GLN-212 residues of CD55, leading to the dissociation of EGFR from the membrane and promoting its translocation to the nucleus. This signaling cascade inhibits p53 activity, thereby causing dysregulation of lipid metabolism and contributing to radioresistance. Understanding this regulatory pathway unveils new therapeutic targets and strategies for addressing radioresistance in rectal cancer.

## Supplementary information

Supplementary Figure

Supplementary Figure Legends

checklist

WB Original Image

## Data Availability

The datasets used and/or analyzed during the current study are available from the corresponding author upon reasonable request. RNA-seq data for HCT116 (GSE319208) were downloaded from the Gene Expression Omnibus (GEO) database (https://www.ncbi.nlm.nih.gov/geo/).
