# Supplementary figures and images for: GDPD5-CD55-EGFR competitive binding axis regulates radioresistance and lipid accumulation in rectal cancer
Source: Cell Death Dis. 2026 Apr 7;17(1):492. doi: 10.1038/s41419-026-08711-3 (PMC13187013; doi:10.1038/s41419-026-08711-3)

**Figure S1**

**A**

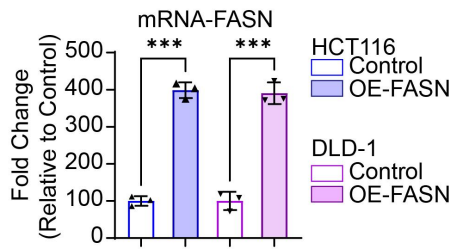

**B**

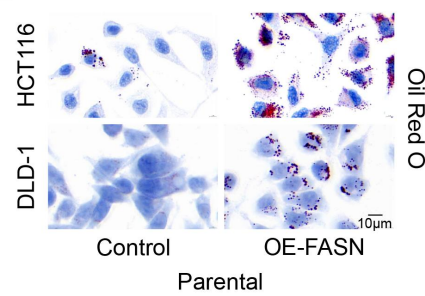

**C**

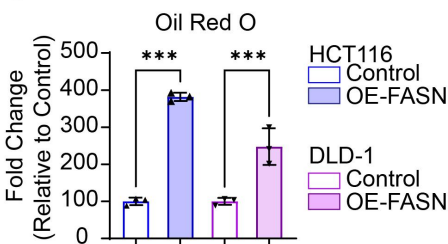

**D**

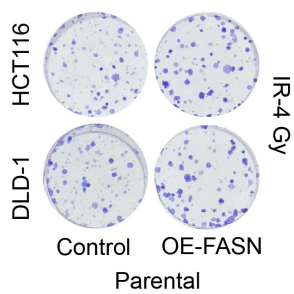

**E**

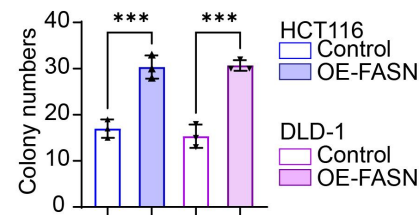

**F**

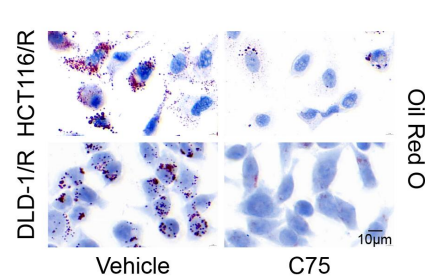

**G**

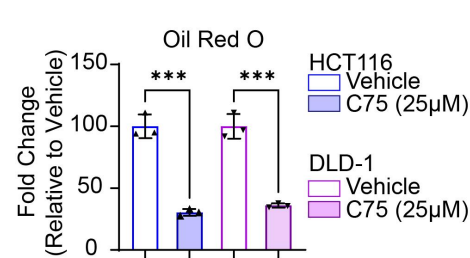

**H**

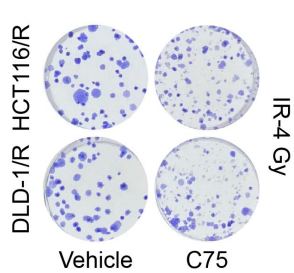

**I**

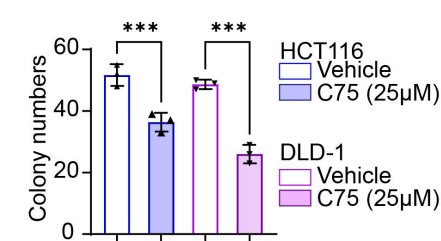

**Figure S2**

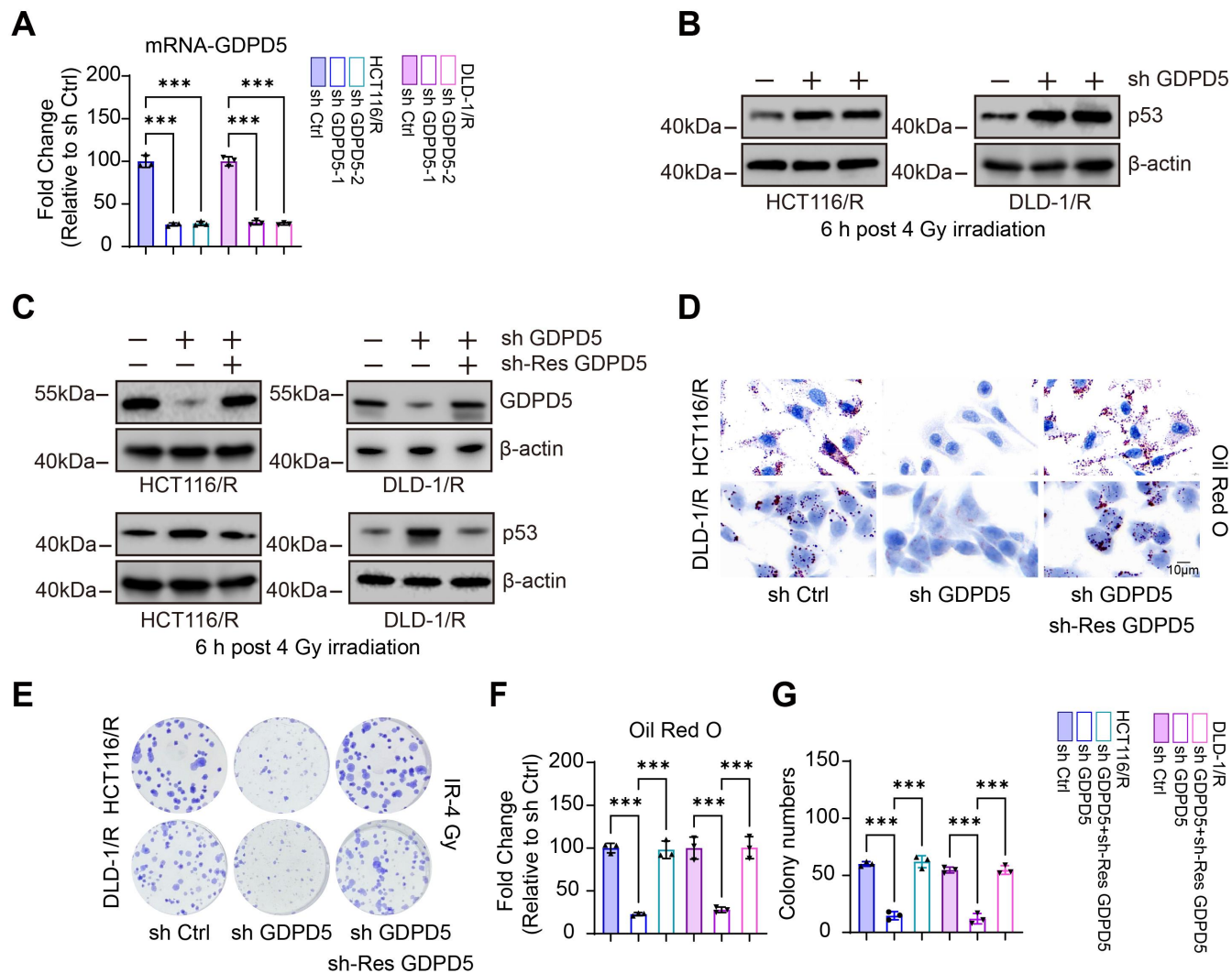

**Figure S3**

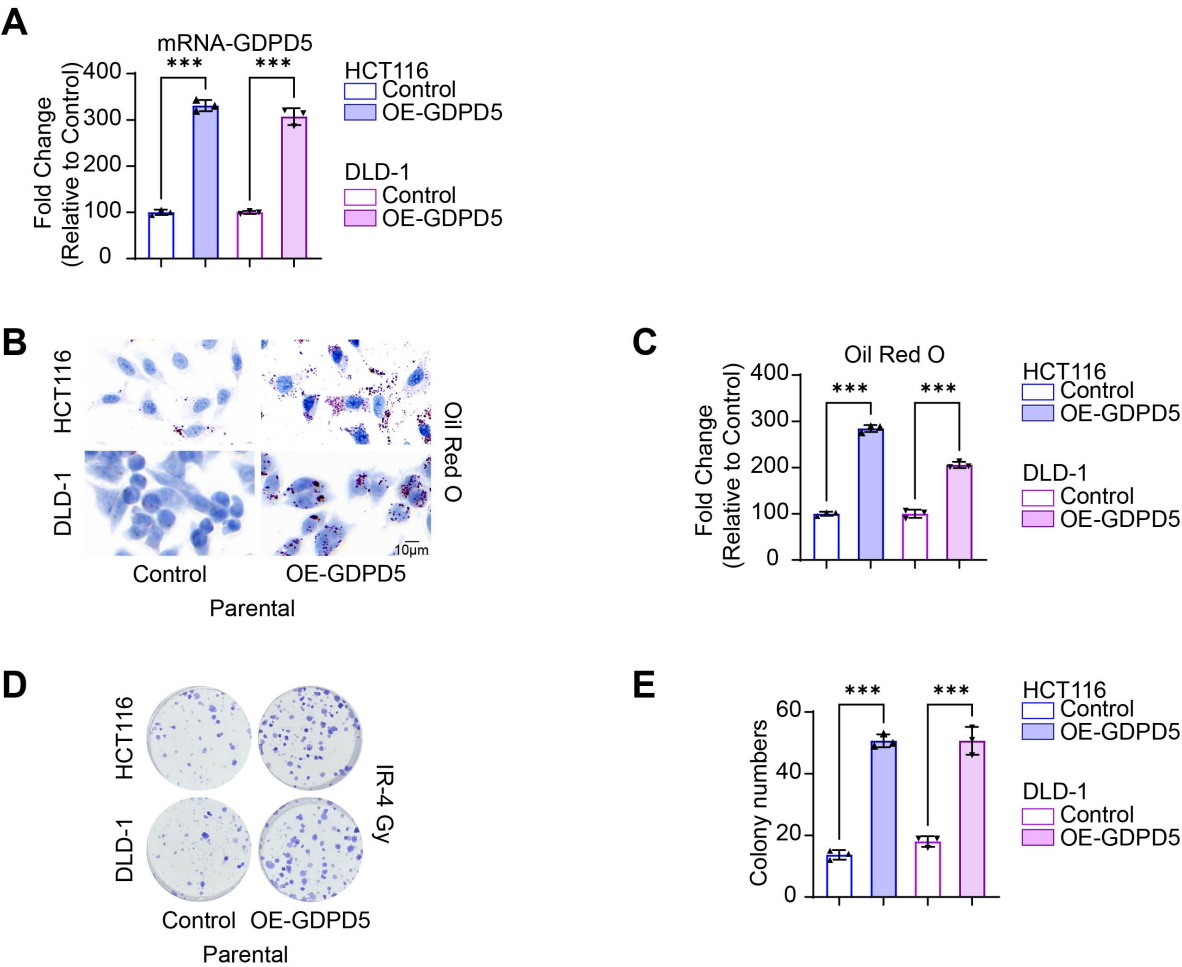

Figure S4

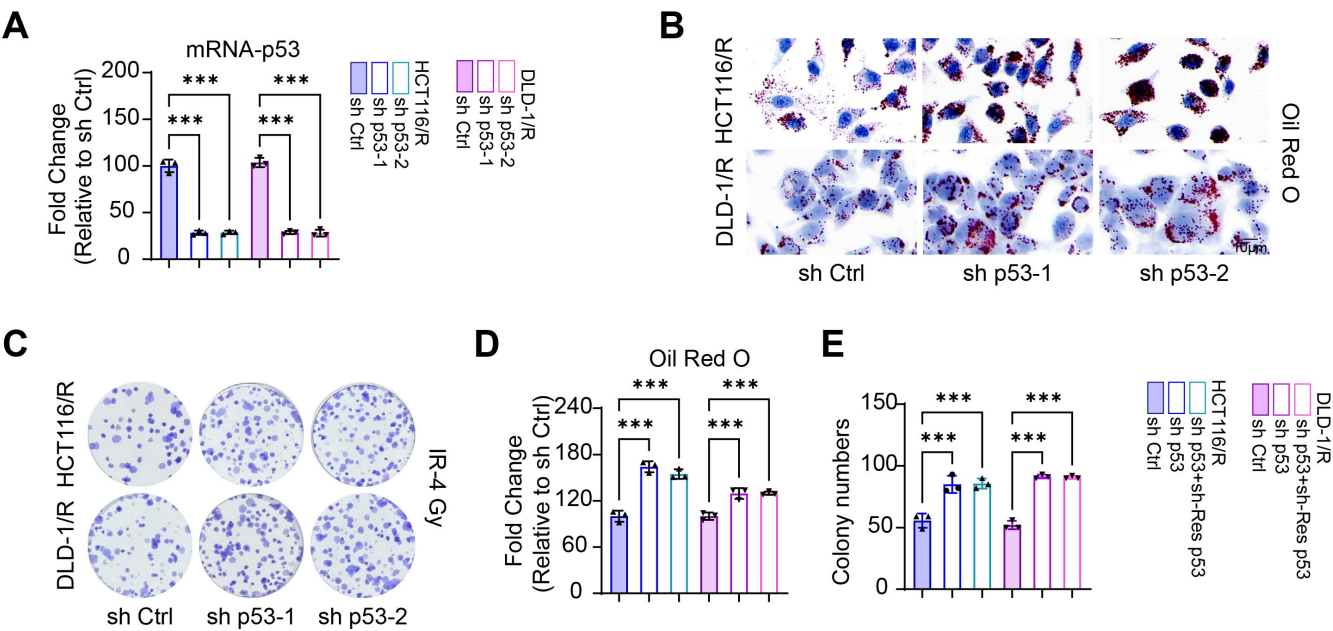

**Figure S5**

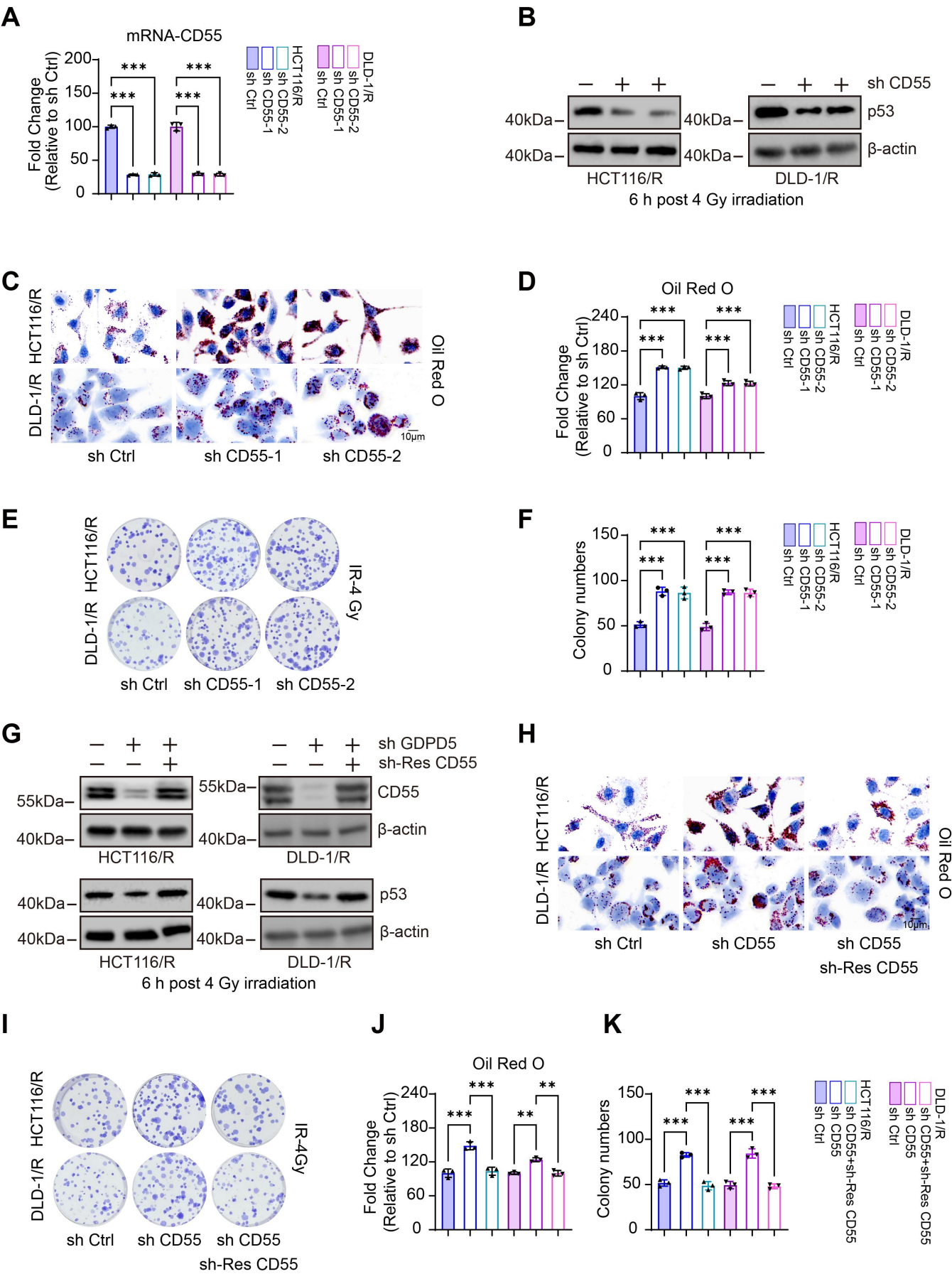

Figure S6

A

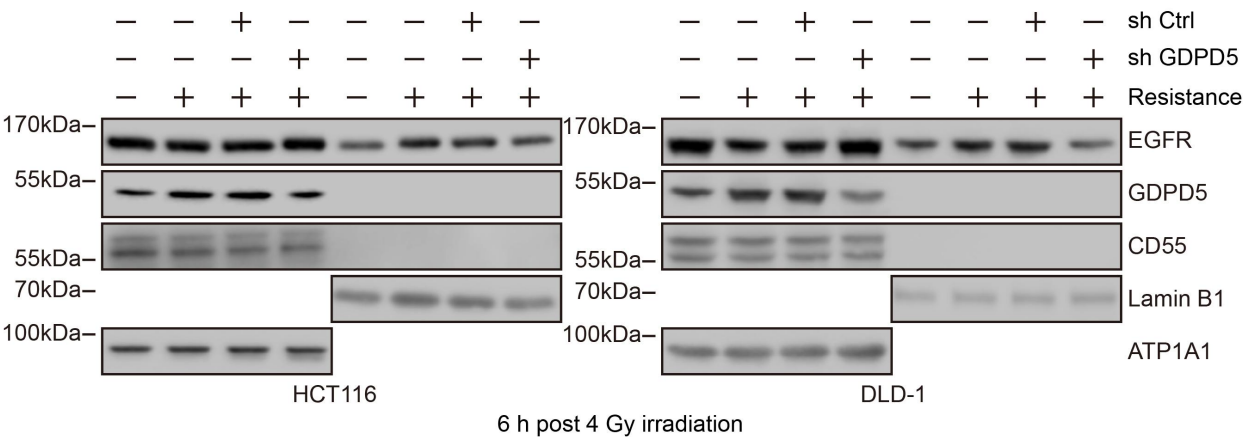

**Figure S7**

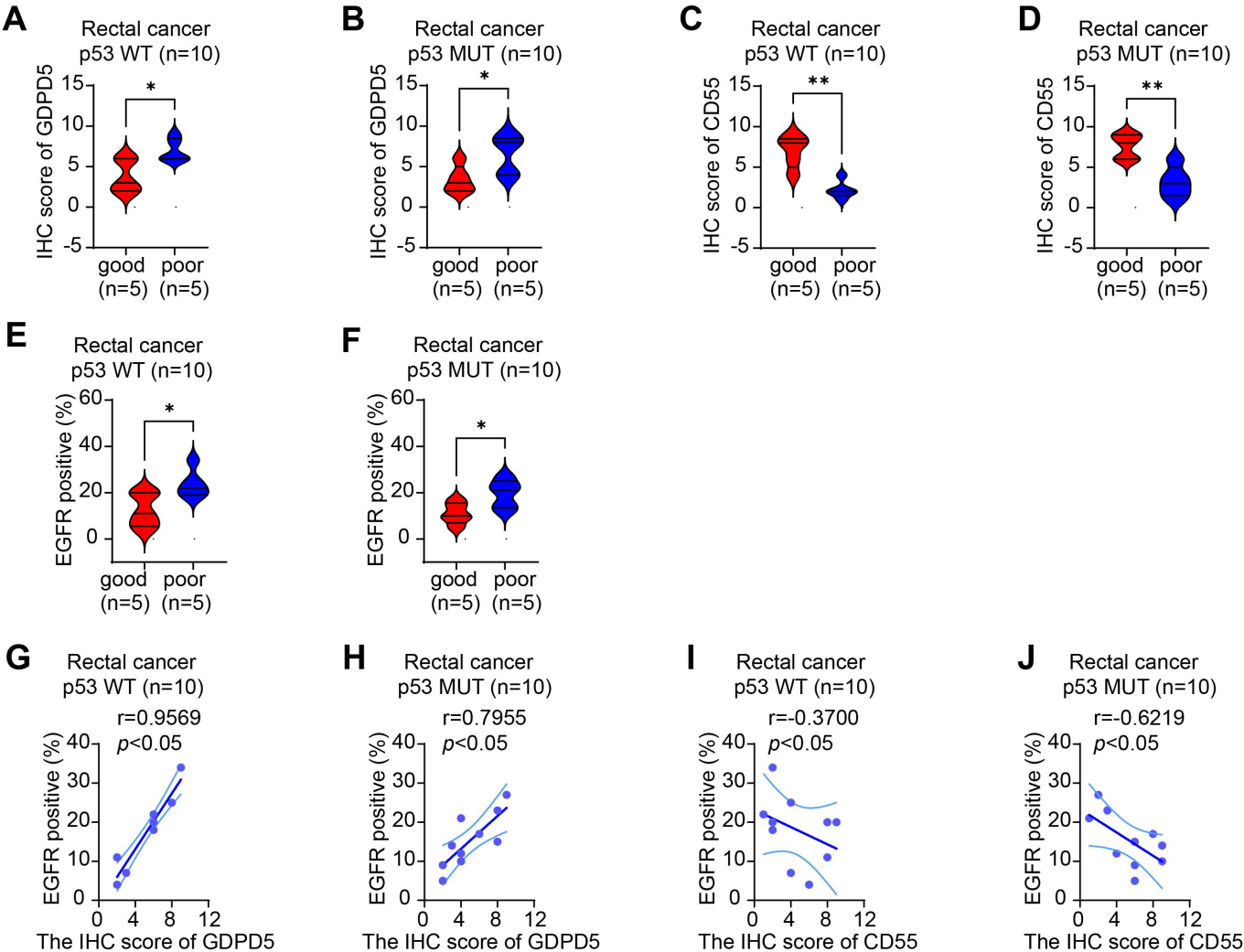

Figure S8

A

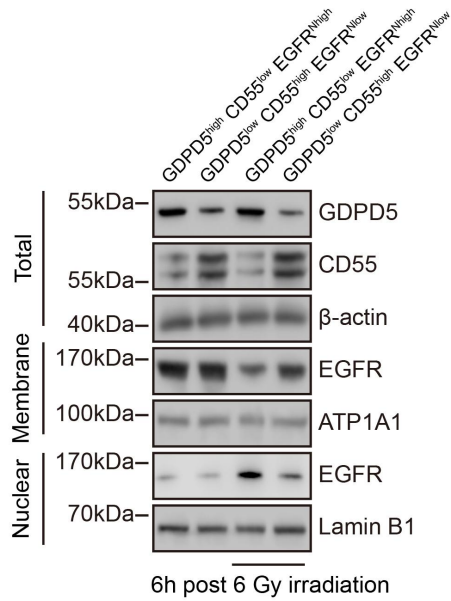

B

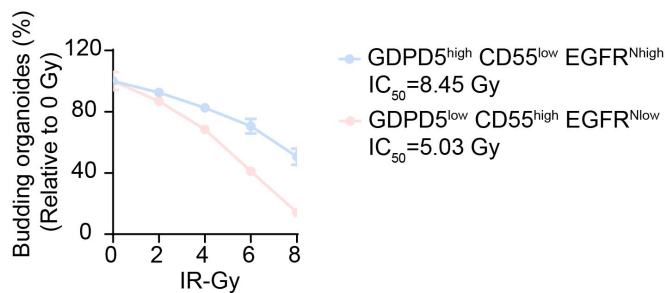

C

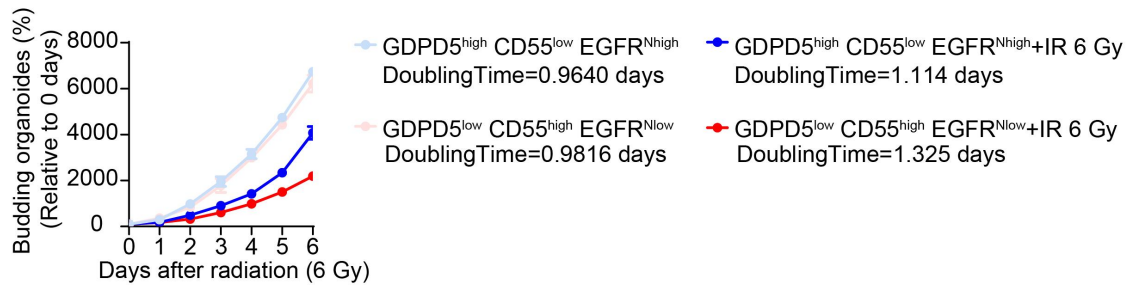

D

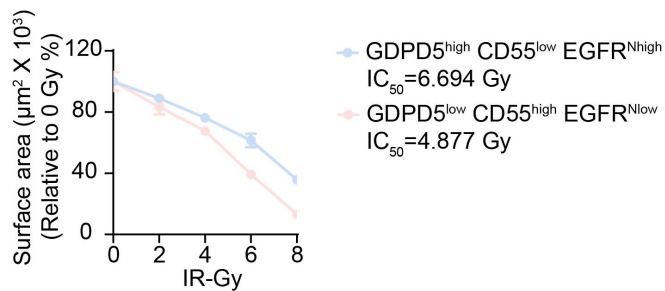

E

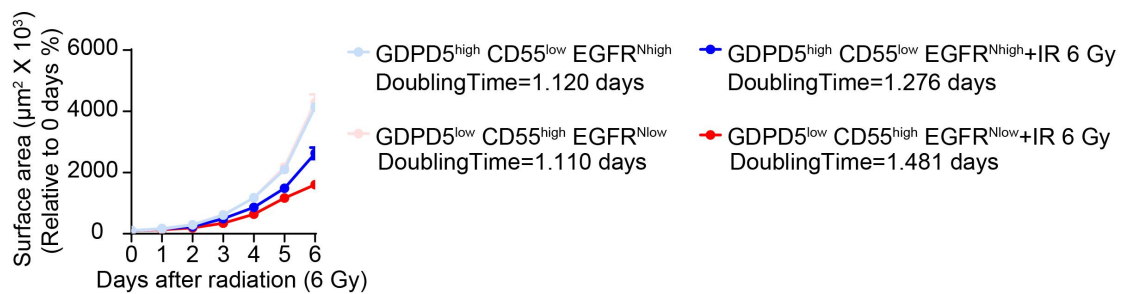

Supplement: Supplementary file 1 — Supplementary Figure [file 41419_2026_8711_MOESM1_ESM.pdf]
