# Supplementary material for: GDPD5-CD55-EGFR competitive binding axis regulates radioresistance and lipid accumulation in rectal cancer
Source: Cell Death Dis. 2026 Apr 7;17(1):492. doi: 10.1038/s41419-026-08711-3 (PMC13187013; doi:10.1038/s41419-026-08711-3)
Supplement: Supplementary file 2 — Supplementary Figure Legends [file 41419_2026_8711_MOESM2_ESM.docx]

Figure S1 The Association Between Lipid Content and Radiation Sensitivity in Cellular Models (A) qPCR experimental validation of FASN overexpression efficiency in parental cell lines. (B-C) Oil Red O staining (scale bar = 10 μm) and quantification of neutral lipids were performed in parental and OE-FASN HCT116 and DLD-1 cell lines. (D-E) Clonogenic assays and statistical analyses were performed on both parental and OE-FASN HCT116 and DLD-1 cell lines. (F-G) Oil Red O staining (scale bar = 10 μm) and quantification of neutral lipids were performed in vehicle and C75 HCT116/R and DLD-1/R cell lines. (H-I) Clonogenic assays and statistical analyses were performed in vehicle and C75 HCT116/R and DLD-1/R cell lines. NS indicates non-significance, **p<0.01; ***p<0.001.

Figure S2 GDPD5 Knockdown and Rescue (A) qPCR experimental validation of GDPD5 shRNA efficiency in HCT116/R and DLD-1/R cell lines. (B) Western blot analysis was also utilized to assess the protein levels of p53 in control versus GDPD5-knockdown radioresistant cell lines. (C) Western blot analysis was also utilized to assess the protein levels of GDPD5 and p53 in control versus, GDPD5-knockdown and GDPD5-rescue radioresistant cell lines. (D, F) Oil Red O staining (scale bar = 10 μm) and quantification of neutral lipids were conducted in control, GDPD5-knockdown and GDPD5-rescue radioresistant cells. (E, H) Clonogenic assays (IR-4 Gy) and statistical analyses were conducted in control, GDPD5-knockdown and GDPD5-rescue radioresistant cells. NS: not significant; **p<0.01; ***p<0.001.

Figure S3 GDPD5 Overexpression (A) qPCR experimental validation of GDPD5 overexpression plasmid efficiency in HCT116 and DLD-1 cell lines. (B, C) Oil Red O staining (scale bar = 10 μm) and quantification of neutral lipids were conducted in control and GDPD5 overexpression HCT116 and DLD-1. (C, E) Clonogenic assays (IR-4 Gy) and statistical analyses were conducted in control and GDPD5 overexpression HCT116 and DLD-1. NS: not significant; **p<0.01; ***p<0.001.

Figure S4 p53 Knockdown (A) qPCR experimental validation of p53 shRNA efficiency in HCT116/R and DLD-1/R cell lines. (B, D) Oil Red O staining (scale bar = 10 μm) and quantification of neutral lipids were conducted in control and p53-knockdown radioresistant cells. (C, E) Clonogenic assays (IR-4 Gy) and statistical analyses were conducted in control and p53-knockdown radioresistant cells. NS: not significant; **p<0.01; ***p<0.001.

Figure S5 CD55 Knockdown and Rescue (A) qPCR experimental validation of CD55 shRNA efficiency in HCT116/R and DLD-1/R cell lines. (B) Western blot analysis was also utilized to assess the protein levels of p53 in control versus CD55-knockdown radioresistant cell lines. (C,D) Oil Red O staining (scale bar = 10 μm) and quantification of neutral lipids were conducted in control and CD55-knockdown radioresistant cells. (E, F) Clonogenic assays (IR-4 Gy) and statistical analyses were conducted in control and CD55-knockdown radioresistant cells. (G) Western blot analysis was also utilized to assess the protein levels of CD55 and p53 in control versus, CD55-knockdown and CD55-rescue radioresistant cell lines. (H, J) Oil Red O staining (scale bar = 10 μm) and quantification of neutral lipids were conducted in control, CD55-knockdown and CD55-rescue radioresistant cells. (I, K) Clonogenic assays (IR-4 Gy) and statistical analyses were conducted in control, CD55-knockdown and CD55-rescue radioresistant cells. NS: not significant; **p<0.01; ***p<0.001.

Figure S6 Subcellular Fractionation Assays (A) Western blot analysis was utilized to assess the protein levels of EGFR, GDPD5 and CD55 in parental, radioresistant, radioresistant + shCtrl and radioresistant + shGDPD5 cell lines in the cell membrane and nucleus.

Figure S7 Analysis of p53 Mutation Status and Associations with GDPD5, CD55, Nuclear EGFR, and Treatment Response. (A-F) Immunohistochemical analysis of GDPD5, CD55, and EGFR expression in rectal cancer tissue samples (n=20 (good=10, p53 WT=5, p53 MUT=5) (poor=10, p53 WT=5, p53 MUT=5)), accompanied by IHC scoring (B-D). (G-J) Correlation analysis of the IHC findings. NS denotes non-significance; *p<0.05; **p<0.01; ***p<0.001.

Figure S8 Radiation Dose and Time Effects on Organoids. (A) Western blot analysis was performed to detect the expression of GDPD5, CD55, and EGFR in cell membranes, nuclei, and total proteins of organoids 6 hours after 6 Gy irradiation. Both GDPD5-low-expressing CD55-high-expressing EGFR-low-nuclear-entry organoids and GDPD5-high-expressing CD55-low-expressing EGFR-high-nuclear-entry organoids underwent statistical analysis of budding rates (B) and surface areas (D) at 6 days post-irradiation following 0, 2, 4, 6, and 8 Gy ionizing radiation. Additionally, measurements were taken at 0, 1, 2, 3, 4, 5, and 6 days post-6 Gy ionizing radiation (C, E).
