# Supplementary material for: GDPD5-CD55-EGFR competitive binding axis regulates radioresistance and lipid accumulation in rectal cancer
Source: Cell Death Dis. 2026 Apr 7;17(1):492. doi: 10.1038/s41419-026-08711-3 (PMC13187013; doi:10.1038/s41419-026-08711-3)

Figure 1F

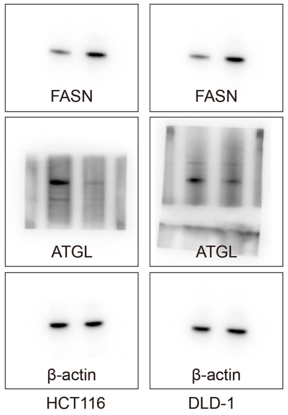

Figure 2B

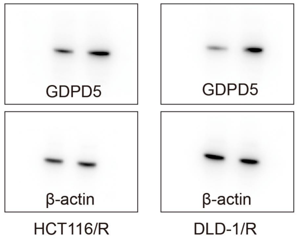

Figure 2C

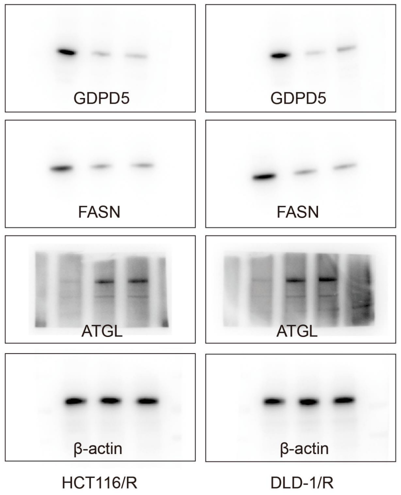

Figure 3E

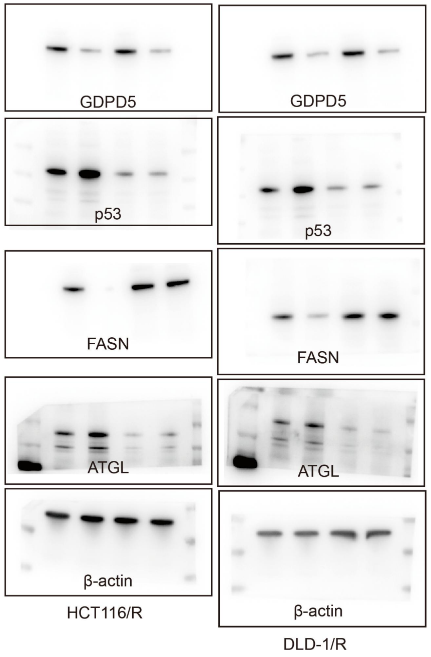

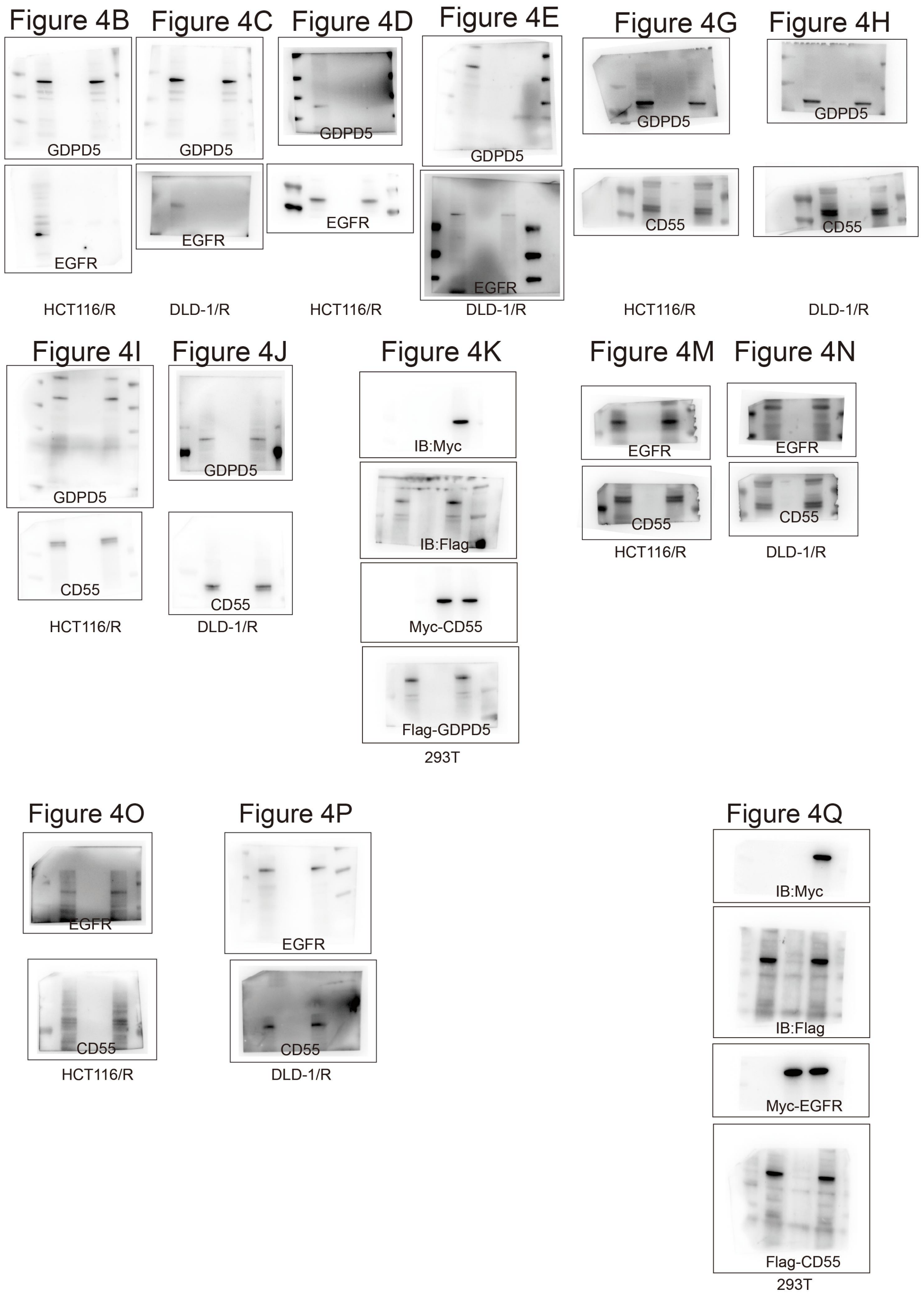
Figure 4B
GDPD5
EGFR
HCT116/R
Figure 4C
GDPD5
EGFR
DLD-1/R
Figure 4D
GDPD5
EGFR
HCT116/R
Figure 4E
GDPD5
EGFR
DLD-1/R
Figure 4G
GDPD5
CD55
HCT116/R
Figure 4H
GDPD5
CD55
DLD-1/R
Figure 4I
GDPD5
CD55
HCT116/R
Figure 4J
GDPD5
CD55
DLD-1/R
Figure 4K
IB:Myc
IB:Flag
Myc-CD55
Flag-GDPD5
293T
Figure 4M
EGFR
CD55
HCT116/R
Figure 4N
EGFR
CD55
DLD-1/R
Figure 4O
EGFR
CD55
HCT116/R
Figure 4P
EGFR
CD55
DLD-1/R
Figure 4Q
IB:Myc
IB:Flag
Myc-EGFR
Flag-CD55
293T

Figure 5C

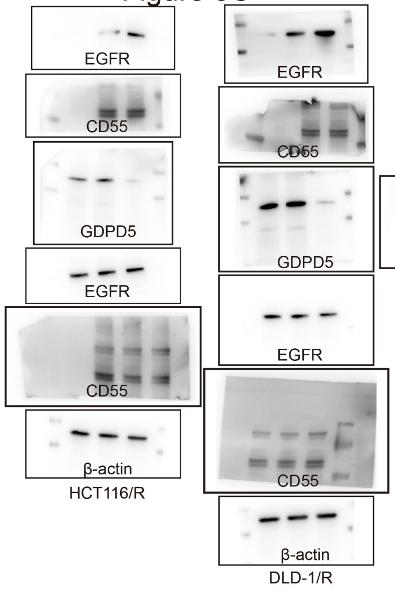

Figure 5D

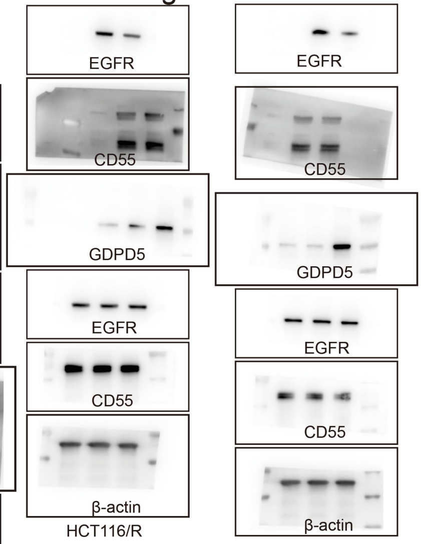

Figure 5E

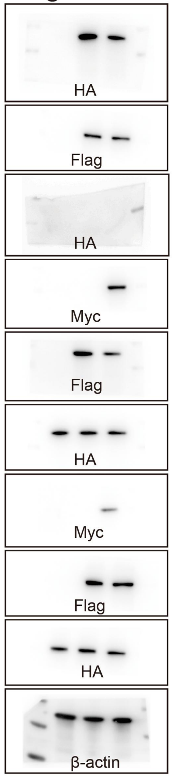

Figure 5F

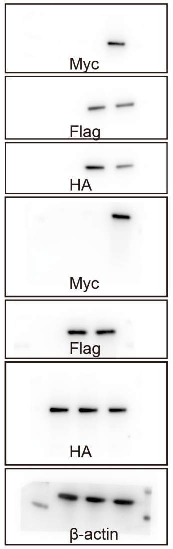

Figure 5G

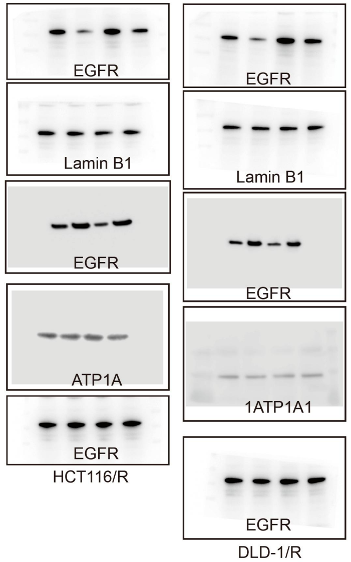

Figure 6F

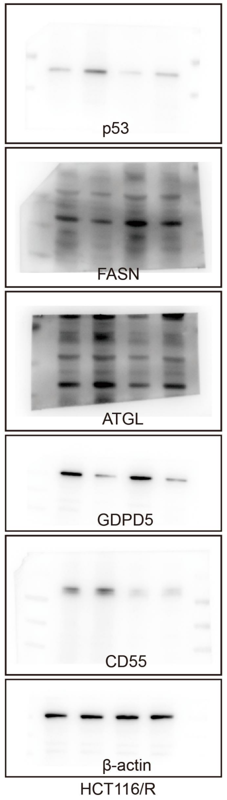
p53
FASN
ATGL
GDPD5
CD55
β-actin
HCT116/R

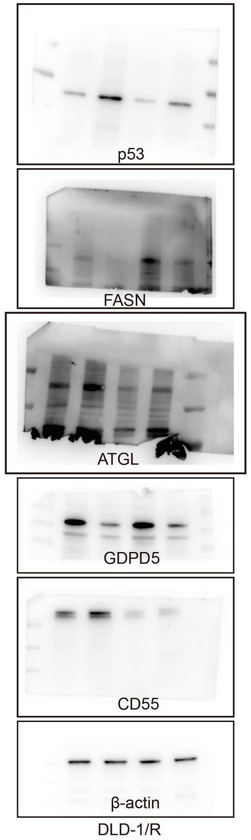
p53
FASN
ATGL
GDPD5
CD55
β-actin
DLD-1/R

Figure S2B

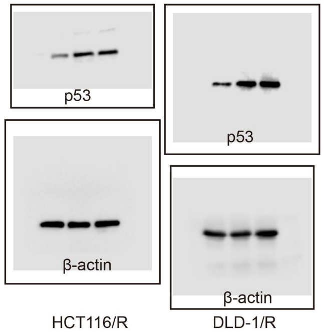

Figure S2C

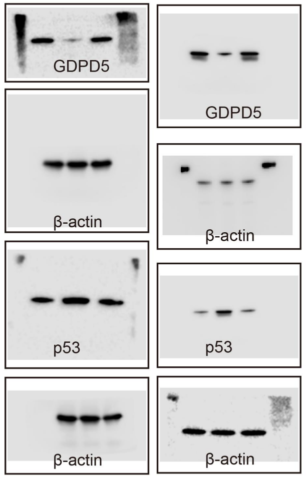

Figure S4B

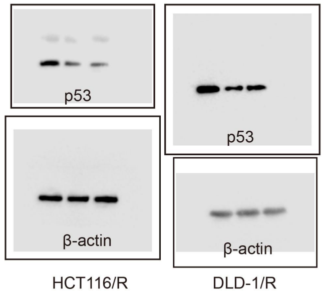

Figure S3C

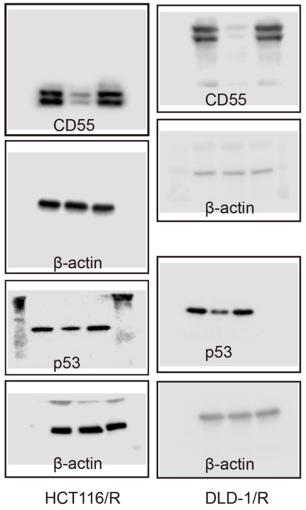

Figure S6A

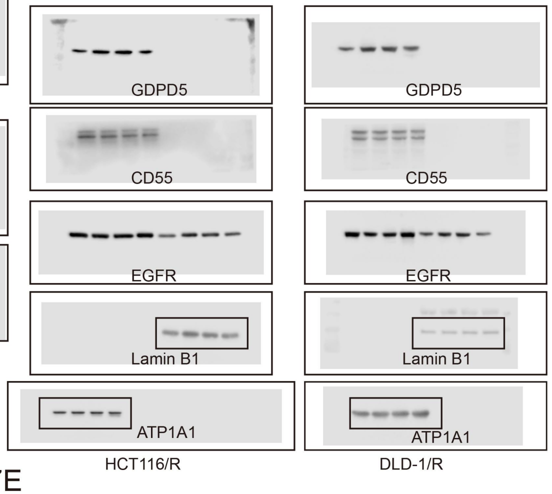

Figure S7E

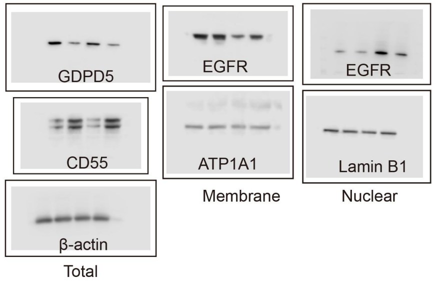

Supplement: Supplementary file 4 — WB Original Image [file 41419_2026_8711_MOESM4_ESM.pdf]
